# Morphological and optical properties of Pd_x_Ag_1-x_ alloy nanoparticles

**DOI:** 10.1080/14686996.2018.1435944

**Published:** 2018-02-22

**Authors:** Sundar Kunwar, Puran Pandey, Mao Sui, Sushil Bastola, Jihoon Lee

**Affiliations:** ^a^ College of Electronics and Information, Kwangwoon University, Seoul, South Korea; ^b^ Institute of Nanoscale Science and Engineering, University of Arkansas, Fayetteville, AR, USA

**Keywords:** PdAg nanoparticles, alloy nanoparticles, solid-state dewetting, bilayer dewetting, surface plasmon, 40 Optical, magnetic and electronic device materials, 106 Metallic materials, 101 Self-assembly / Self-organized materials, 208 Sensors and actuators; 209 Solar cell / Photovoltaics, 212 Surface and interfaces, 306 Thin film / Coatings, 502 Electron spectroscopy, 503 TEM, STEM, SEM, 504 X-ray / Neutron diffraction and scattering

## Abstract

Alloy nanoparticles (NPs) can offer a wide range of opportunities for various applications due to their composition and structure dependent properties such as multifunctionality, electronic heterogeneity, site-specific response, and multiple plasmon resonance bands. In this work, the fabrication of self-assembled Pd_x_Ag_1-x_ NPs alloy nanostructures with distinct size, density, shape, and composition is demonstrated via the solid-state dewetting of sputtered Pd/Ag thin films on c-plane sapphire. The initial stage of bilayer dewetting exhibits the nucleation of voids, followed by the expansion of voids and cluster breakdown and finally shape transformation along with the temperature control. Bilayer composition shows a substantial influence on the dewetting such that the overall dewetting is enhanced along with the increased Ag composition, i.e. Pd_0.25_Ag_0.75_ > Pd_0.5_Ag_0.5_ > Pd_0.75_Ag_0.25_. On the other hand, the size and density of NPs can be efficiently controlled by varying the initial thickness of bilayers. Reflectance peaks in UV and near-infrared (NIR) regions and a wide absorption band in the visible region arisen from the surface plasmon resonance are observed in reflectance spectra. The peak intensity depends on the composition of Pd_x_Ag_1-x_ NPs and the NIR peaks gradually blue-shift with the size decrement.

## Graphical abstract

**Figure:**
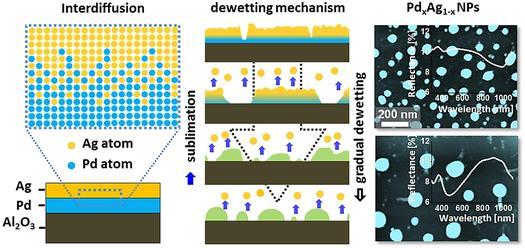

## 1. Introduction

Metallic alloy NPs have gained increasing research attention due to the added possibility of tuning optical, electronic and catalytic properties by controlling the alloy composition and surface morphology [1–3]. More specially, the multi-metallic alloy NPs promisingly extend the range of applications due to their bi-functionality, electronic heterogeneity, site specific response and multiple plasmon resonance band that could not be achieved with only monometallic NPs [4–8]. By the appropriate choice of alloy materials, compositions and structures, further opportunities can be found in several fields including electronics, photonics, energy, catalysis, sensing, and medicine [9–14]. For example, gold–palladium (Au–Pd) alloy NPs have shown a profound enhancement in the catalytic activity due to the electronic heterogeneity of alloy NPs that yields wide distribution of Pd sites and production of light absorbed electrons by the localized surface plasmon resonance (LSPR) of Au [4]. At the same time, palladium and silver are important materials for catalysis, oxygen reduction, hydrogen synthesis, and storage devices [15–20] due to the high hydrogen affinity and excellent catalytic activity of Pd and electrons confinement by surface plasmon resonance of Ag. In aforementioned applications, the chemical reactivity, optical coupling by LSPR and charge transfer rates mainly depends on the electronic interaction between NPs and supporting material, which can be appropriately modulated by the control of structure and composition of NPs. Therefore, the fabrication of Pd–Ag alloy NPs by the solid stage dewetting on sapphire (0 0 0 1) and the systematic study on their structural and optical properties can be an important foundation for many potential applications, which is not yet reported up to date. In this work, the Pd_x_Ag_1-x_ alloy NPs are fabricated on c-plane sapphire (0 0 0 1) via the solid-state dewetting approach and the evolution of Pd_x_Ag_1-x_ NPs from the Pd/Ag bilayer is demonstrated by the systematic control of various growth parameters. Depending upon the variation of annealing temperature, film thickness, and composition of bilayers, various surface morphologies, size and density of Pd_x_Ag_1-x_ NPs are demonstrated. The temperature induced surface phenomena such as surface and interface diffusion, agglomeration, surface and interface energy minimization and equilibrium state of the thermodynamic system are correlated with the evolution of Pd_x_Ag_1-x_ NPs. The alloy NPs are characterized by reflectance spectroscopy and the formation and shift of reflectance peaks are discussed based on LSPR effect of alloy NPs. In addition, the Raman spectra analysis is performed to probe the strain effect and crystal properties of sapphire after Pd_x_Ag_1-x_ NPs fabrication.

## 2. Experimental section

In this work, single side-polished 430 μm thick sapphire (0 0 0 1) wafer with ±0.1° off-axis was used as a substrate (iNExus Inc, Seoul, South Korea). Prior to the deposition of thin films, the substrates were degassed in a pulsed laser deposition (PLD) chamber at 600 °C under 1 × 10^−4^ Torr for 30 min. During the high temperature treatment, the surface contaminants and gaseous particles were removed and substrates were ready for the deposition. The surface morphology of bare sapphire after degassing showed very smooth texture with steps below 0.5 nm as shown in the Figure S1. In addition, the Raman spectra revealed six characteristic vibration modes of natural sapphire and reflectance spectra exhibited almost uniform response over 300–1100 nm wavelength. Then, Pd and Ag bilayers (Ag top layer) were sequentially deposited on sapphire (0 0 0 1) in a sputtering chamber. Both layers were deposited under identical conditions: i.e. 0.05 nm s^−1^, 3 mA and 1 × 10^−1^ Torr were growth rate, ionization current and chamber pressure, respectively. The thickness of bilayer was controlled by the deposition time and the total thickness is sum of individual thickness of Pd and Ag layer. Three distinct bilayer compositions were prepared by varying the thickness of Pd and Ag layer such as: (i) Pd_0.25_Ag_0.75_ (75% of Ag), (ii) Pd_0.5_Ag_0.5_, and (iii) Pd_0.75_Ag_0.25_. As deposited bilayers were examined by atomic force microscope (AFM) as displayed in Figure S2, which shows a gradually enhanced surface roughness and grain formation with the increased Ag thickness. Samples with 15 nm total thickness were annealed between 400 and 900 °C for annealing temperature effect for all three bilayer compositions. Another series of samples with variable total thickness between 1 and 30 nm were fabricated at 850 °C for all three compositions. The annealing was performed in a PLD chamber with the linear ramping of temperature by 4 °C s^−1^ under 1 × 10^−4^ Torr in order to reach the target temperature. The annealing process was controlled by a computer program to ensure the consistency. After reaching each target temperature, fixed annealing duration of 120 s was allocated to each sample. To finish the growth, each sample was kept in vacuum until the temperature reach to the ambient after turning off the heating system. The surface morphologies of as-fabricated samples were characterized by an atomic force microscope (AFM-NC, Park Systems XE – 70, Suwon, South Korea) and scanning electron microscope (SEM, COXEM CX-200, Daejeon, South Korea). Same batch of AFM tips NSC16/AIBS with drive frequency of ~270 kHz was used. Furthermore, the elemental composition of alloy NPs was measured by energy-dispersive X-ray spectroscope (EDS) from Thermo Fisher Scientific (Noran System 7, USA). The optical properties of Pd–Ag alloy nanostructures on sapphire (0 0 0 1) were investigated via reflectance spectra using a CCD spectrograph, various light sources (deuterium and halogen) and UNIRAM II system from (UNiNanoTech Co. Ltd., Yongin, South Korea). In addition, the substrate properties such as stress effect and crystallinity were investigated by the measurement of Raman spectra using 532 nm laser.

## 3. Results and discussion

**Figure 1.**
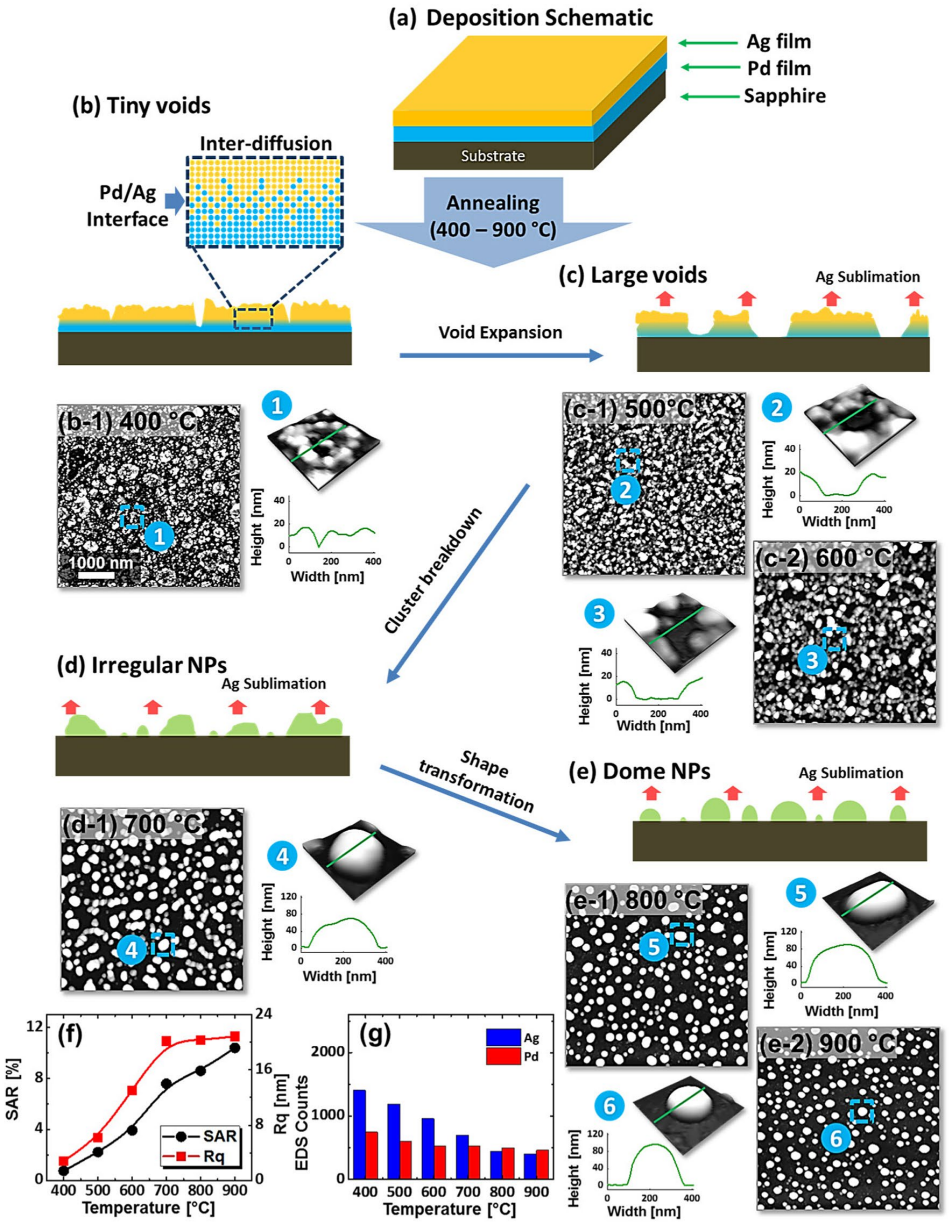
Evolution of Pd_0.25_Ag_0.75_ nanoparticles (NPs, total thickness 15 nm) on sapphire (0 0 0 1) with annealing temperature between 400 and 900 °C. (a) Schematic of Pd/Ag bilayer deposition. (b)–(g) Schematics of surface phenomena and corresponding AFM top-views of 5 × 5 μm^2^ in (b-1)–(e-2). Insets show the 3D side-views and line profiles of typical regions. (f) RMS roughness (Rq), surface area ratio (SAR) and (g) EDS counts with respect to the temperature.

**Figure 2.**
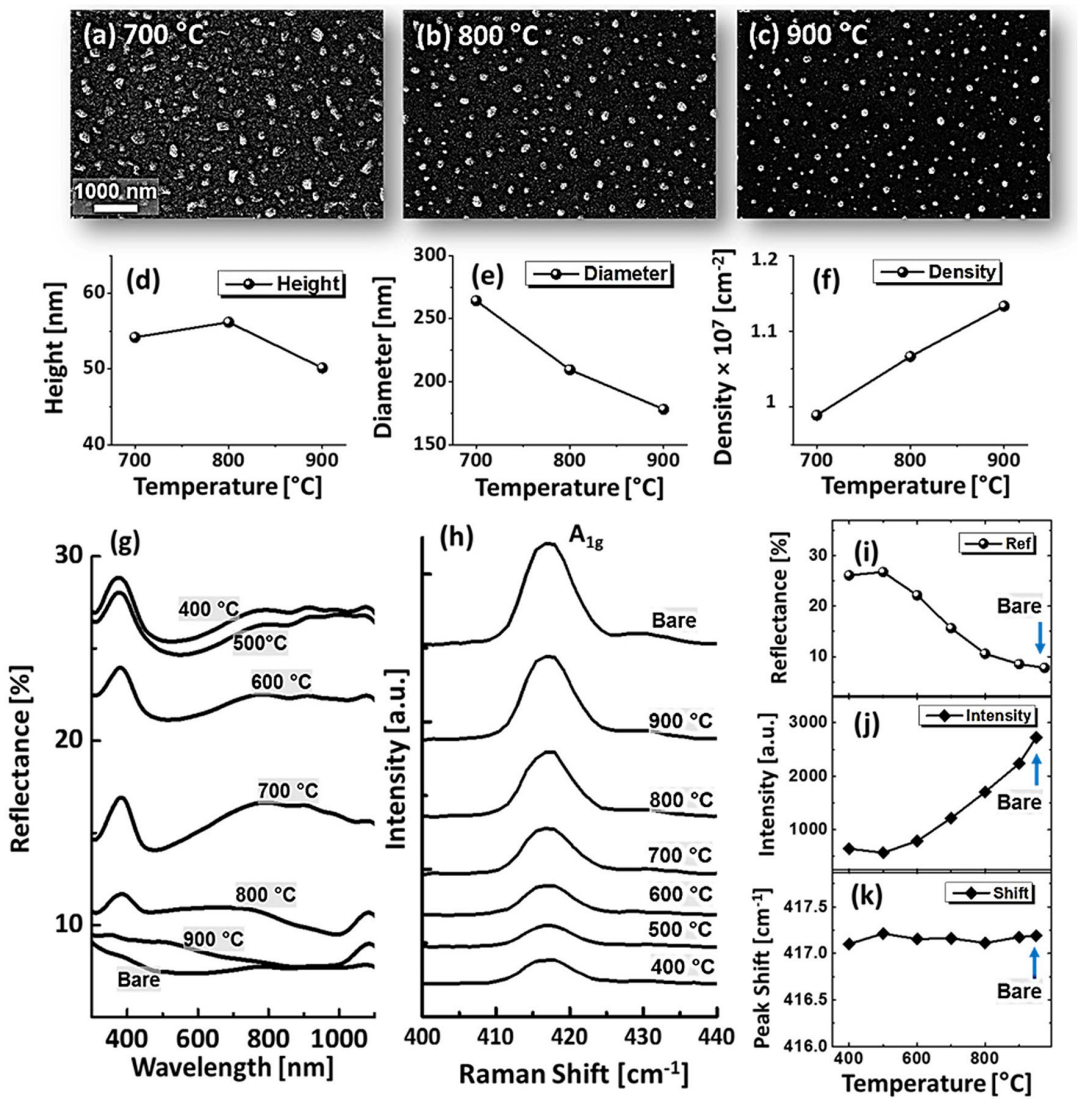
(a)–(c) SEM images of alloy NPs, annealed between 700 and 900 °C for 120 s with 15 nm total thickness. (d)–(f) Average height, diameter and density of the alloy NPs. (g) Reflectance spectra of Pd-Ag alloy NPs on sapphire (0 0 0 1). (h) Raman spectra of A_1g_ vibration mode at ~417 cm^−1^ of each sample. (i) Average reflectance, (j) Raman intensity, and (k) Raman peak shift with respect to the temperature.

Figure 1 shows the evolution of Pd_x_Ag_1-x_ alloy nanostructures from Ag/Pd bilayers by the subsequent annealing between 400 and 900 °C for 120 s. The total thickness of bilayers is 15 nm that consist of 3.75 nm Pd and 11.25 nm Ag (Pd_0.25_Ag_0.75_) as shown in deposition schematic in Figure 1(a). The growth of alloy NPs can be divided into four stages such as: tiny voids, large voids (void expansion), irregular NPs (cluster breakdown) and spherical (shape transformation) NPs as shown in Figure 1(b)–(e). In addition, the corresponding AFM images and line profiles demonstrate the surface phenomena and morphological transitions. Initially, metal films deposited on substrates at ambient temperature are unstable or metastable, which normally dewet into isolated particles along with an appropriate annealing to gain thermodynamic stability with the lowest surface energy. In case of bilayer film deposition, the interfacial layer can be formed between Ag/Pd layers which contains both Pd and Ag atoms. By the annealing of such bilayer films, the surface and interface diffusion (alloying) can occur simultaneously through the surface and interface of the bilayers as shown in the schematic in Figure 1(b). The diffusivity of atoms (*D*_*s*_) is expressed by the relation: $D_{s}\alpha \exp\left(-\frac{E_{\text{ai}}}{\mathrm{kT}}\right)$, where, *E*_ai_ is the activation energy, *K* is the Boltzmann constant, and *T* is the annealing temperature [21,22]. From the equation, the diffusivity of atoms directly depends on the temperature of the system. Meanwhile, the sputter-deposited bilayers possess numerous low energy sites such as at steps, grain boundaries, vacancies and defects, from where tiny pinholes or voids start to nucleate owing to the diffusion of atoms at favorable temperature. From the surface morphology of as-deposited Ag/Pd bilayer, it consisted of grains and defects which can be due to the natural instability and stronger binding of atoms. Therefore, upon annealing, the dewetting of metal thin film can be initiated by the heterogeneous nucleation at those low energy sites. Once the void are formed, the gradual detachment of thin film from substrate occurs due to capillary forces developed in the void rims as expressed by the relation: $v_{h}=\frac{2D_{s}v_{s}\Omega^{2}\gamma_{\mathrm{fv}}}{kTh^{3}}$, where, $v_{h}$is the growth rate of voids, *v*_*s*_ is the surface density of atoms, *Ω* is the atomic volume of the material, *γ*_*fv*_ is the surface energy of the film, and *h* is the film thickness [23]. Therefore, along with the temperature increment, voids can grow larger by merging the nearby ones as shown in the schematic in Figure 1(c). Correspondingly, atomic inter-mixing can be enhanced through the interface of Pd/Ag bilayers so that the alloyed layer can be enlarged [24–26]. The dewetting of bilayers advances along with the enhanced diffusion, alloying, void growth, and atomic accumulation, resulting in large clusters that finally fragment into the isolated NPs due to the Rayleigh-like instability [27]. Since both Pd and Ag metals are completely soluble in the quasi-solid state, the resulting nanostructures can be consisted of well mixed Pd and Ag atoms for all compositions of Ag/Pd bilayers [28,29]. Based on the previous studies, mono-metallic Ag films were found to dewet very well even at 400 °C resulting in the dome shaped NPs for a wide deposition range whereas Pd thin films showed distinguishable NPs only above 650 °C [9,30]. The dewetting extent vary for different materials due to the difference in parameters such as: diffusivity of materials, chemical potentials, surface and interface energies, atomic size and vacancy concentration. However, the overall dewetting sequence can be comparable such as: nucleation of voids, void growth and finally fragmentation of cluster into the isolated particles as illustrated in the schematic in Figure 1 [23,31]. In case of Ag/Pd bilayers, the initial dewetting was observed at 400 °C as the surface texture became rougher with the formation of tiny voids or pinholes. The overall surface morphology can be observed from the AFM top-views in Figure 1(b-1) and the corresponding enlarged 3D side-view with the line-profile show the detail. The void depth was up to 12 nm whereas the tiny grains on top were 5–10 nm in height. Since the Ag has higher surface diffusivity and low surface energy than the Pd, the Ag atoms can diffuse longer even at relatively low temperature (400 °C) and agglomerate on top layer resulting in the tiny granular structures [32]. Meanwhile, at the interface of Pd and Ag bilayer, the range of inter-mixing can be extended with temperature as discussed. However, the inter-mixing may not be completed due to the lower temperature and short annealing duration. Once pinholes are formed on the Pd layers, Ag atoms can get in, which likely enhances the overall inter-mixing of the bilayers owing to the high diffusivity of Ag. With the increase in temperature, voids were grown further exposing substrate surface at 500 and 600 °C, which can be attributed to the accumulation of the diffusing atoms due to the stronger binding between metallic atoms based on the Volmer-Weber growth [33]. The void growth result in the formation of branched nanoclusters along with the large increment in the dimensions such as; the void width was enlarged up to 100–200 nm while nanoclusters acquired height of ~40 nm. Meanwhile, the Ag sublimation was observed above 500 °C as portrayed by the EDS counts of Pd and Ag at each stage of annealing temperature as shown in Figure 1(i). The rate of Ag sublimation directly depends on the temperature and equilibrium vapor pressure of the system as given by the relation: *R*_*s*_ = (3.513 × 1022) (*T M*_Ag_)^−1/2^ × *P*_eq_, where the *T*, *M*_Ag_ and *P*_eq_ are the annealing temperature, molecular weight of Ag and equilibrium vapor pressure [34]. Therefore, the Ag sublimation escalates exponentially with temperature, which can be clearly seen from the reduced Ag counts. The EDS counts for Ag were gradually dropped, whereas Pd counts were almost similar as shown in the Figure 1(i). Drastic evolution of surface morphology occurred at 700 °C, when the connected clusters were completely fragmented into the isolated-irregular NPs as shown in Figure 1(d). By increasing the annealing temperature up to 900 °C, the isolated alloy NPs were transformed into semi-spherical/dome configuration as shown in Figure 1(e). The shape transformation can be the consequence of attaining equilibrium crystal shape with isotropic energy distribution and hence the overall surface energy can be further reduced toward the thermodynamic stability [30]. Furthermore, the samples are studied on the basis of RMS roughness (Rq) and surface area ratio (SAR). The Rq was consistently increased with temperature up to 700 °C and remained comparable between 700 and 900 °C. Similarly, the SAR was gradually increased with temperature along with the evolution of voids, connected clusters and isolated NPs as the average surface of the isolated NPs is higher than the layer. The large-scale SEM images and average height, diameter and density of dome shaped Pd–Ag alloy NPs between 700 and 900 °C are shown in Figure 2(a)–(f). The average height was slightly increased from ~54 to 56 nm between 700 and 800 °C and decreased to ~50 nm at 900 °C. In terms of average diameter, it was gradually reduced from ~364 to 178 nm whereas the average density was mildly increased from 0.99 × 10^7^ to 1.13 × 10^7^ cm^−2^. The decrease in the dimension of alloy NPs at higher temperature can be attributed to the Ag sublimation as discussed.

The optical properties of Pd–Ag alloy NPs on sapphire (0 0 0 1) were probed by the reflectance spectra as a function of wavelength between 300 and 1100 nm as shown in Figure 2(g). Bare sapphire exhibited ~8% average reflectance with no discernable peaks and valleys. Generally, the average reflectance with Pd–Ag alloy NPs was higher than that of the bare sapphire as shown in Figure 2(i). The dewetting of bilayer yields the formation of voids, elongated, and irregular nanoclusters and semi-spherical alloy NPs, which correspondingly alter the reflectance behavior based on the surface morphology. As both Pd and Ag are highly reflective materials, an increased reflectance can be observed for a higher surface coverage and vice versa. Furthermore, the shape, size, arrangement as well as composition of alloy NPs can determine the wavelength for the surface plasmon resonance [35]. A peak at ~380 nm and wide shoulder in the NIR region were commonly observed up to 700 °C. Meanwhile, the reflectance was suppressed in the visible region (~440–600 nm) creating a wide dip and the dip was gradually contracted and slightly left shifted along with the formation of spherical alloy NPs. The dip formation in the visible band can be the absorption or scattering enhancement by the surface plasmon resonance induced by the Pd-Ag alloy NPs [36,37]. The UV peaks and NIR shoulder were strongly attenuated and the visible-range dip disappeared after annealing at 800 or 900 °C, likely due to the enhanced sublimation of Ag atoms as discussed above. Pure Ag NPs exhibited a sharp quadrupolar resonance peak (UV region) and a dipolar resonance peak (visible to NIR) with narrow absorption band in visible region, whereas Pd NPs exhibited weaker peaks but much wider absorption enhancement in visible region [9,36]. In contrast, Pd–Ag alloy NPs demonstrated intermediated peak intensity and absorption band, which can be attributed to the alloy composition of NPs. Furthermore, the Raman spectra were measured by the excitation of 532 nm laser at 220 mW. Each sample exhibited six Raman bands as shown in Figure S4 due to the A_1g_ vibration mode at ~416.53 cm^−1^ and *E*_*g*_ vibration modes at ~378.24, 446.83, 575.78, and 749.65 cm^−1^ [38]. The intense A_1g_ vibration mode shown in Figure 2(h) was utilized to characterize the samples in term of peak intensity and shift. As seen in Figure 2(i), the intensity was very low at 400 and 500 °C which can be attributed to the large surface coverage of alloy NPs that can significantly absorb incident photon as suggested by the dip in the visible region of reflectance spectra. The Raman intensity was gradually increased along with the void expansion and formation of isolated nanostructures. The intensity variation showed the inverse relationship with average surface coverage of alloy NPs. In case of peak position shift, it was slightly left shift (<0.5 cm^−1^) as compared to the bare sapphire as shown in the Figure 2(k) which can be the consequence of strain developed between the alloy NPs and the substrate molecules [39].

**Figure 3.**
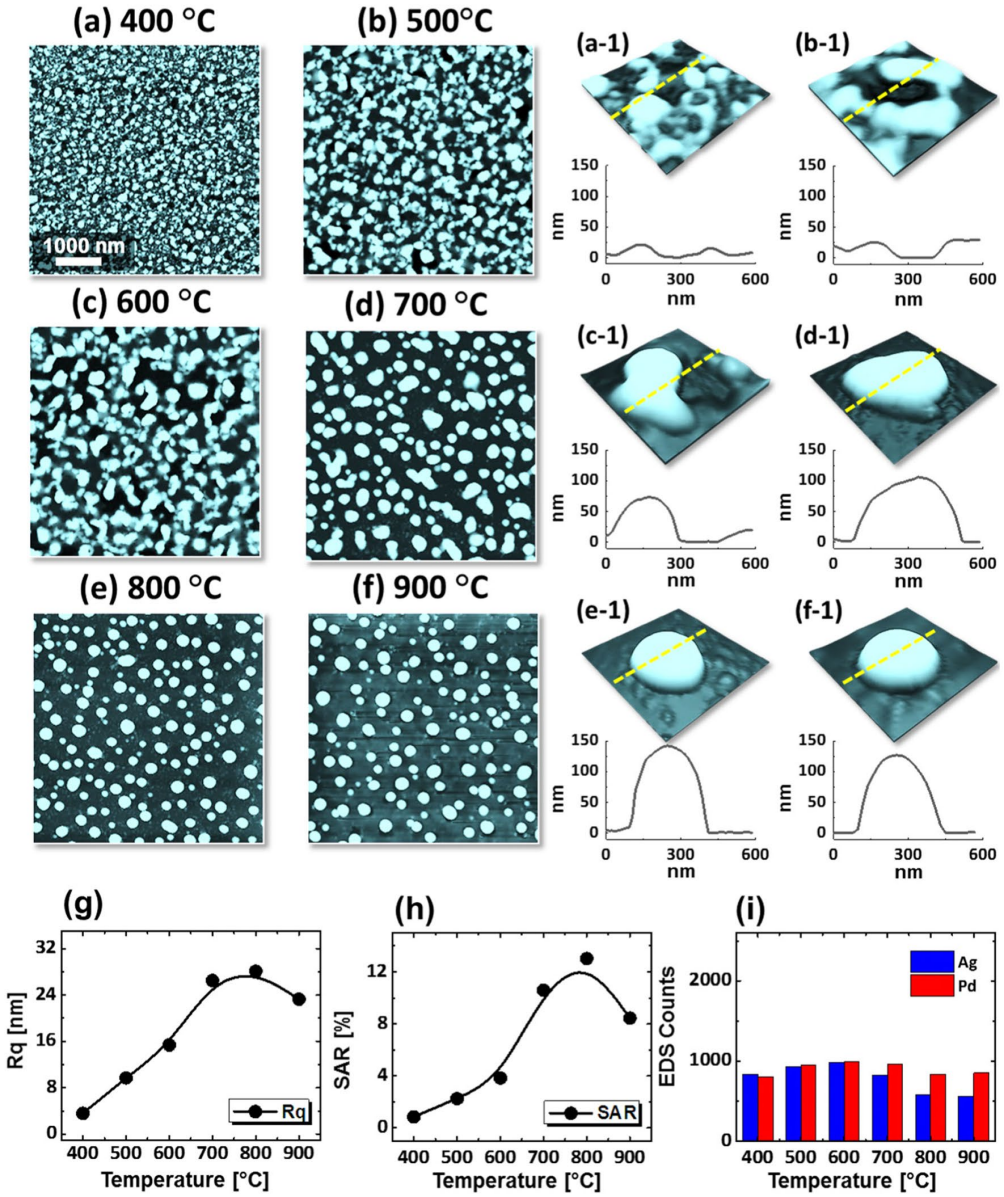
Fabrication of alloy NPs on sapphire (0 0 0 1) with bilayer composition Pd_0.5_Ag_0.5_ and 15 nm total thickness, annealed between 400 and 900 °C for 120 s. (a)–(f) AFM top-views of 5 × 5 μm^2^. (a-1)–(f-1) 3D side-views of typical structures in (a)–(f) and cross-sectional line profiles. (g)–(i) Rq, SAR and EDS count summary with respect to the temperature.

**Figure 4.**
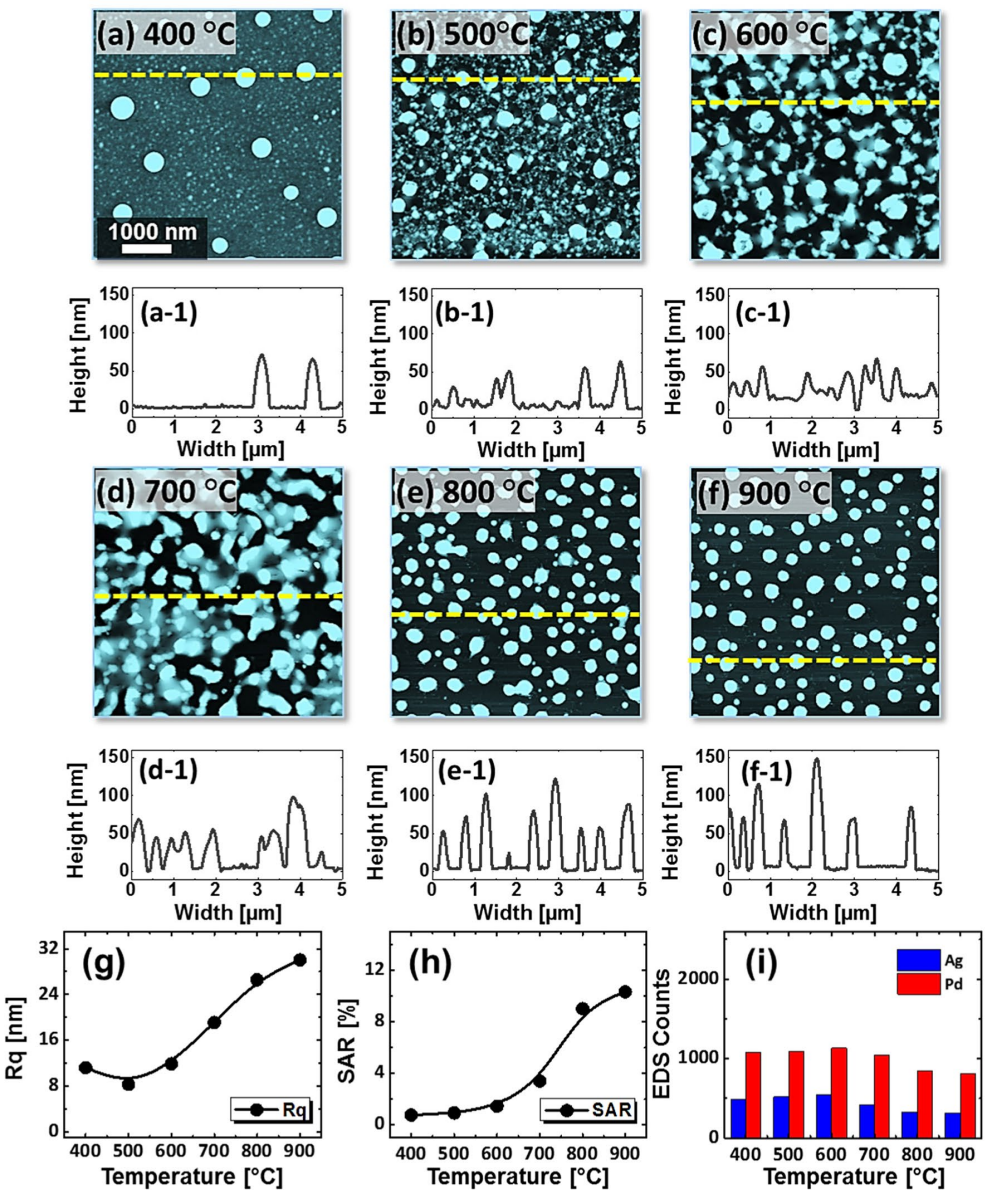
Pd_0.75_Ag_0.25_ NPs on sapphire (0 0 0 1) annealed between 400 and 900 °C for 120 s with 15 nm total thickness. (a)–(f) AFM top-views of 5 × 5 μm^2^. (a-1)–(f-1) Cross-sectional line profiles. (g) Rq, (h) SAR and (i) EDS count with respect to the temperature variation.

**Figure 5.**
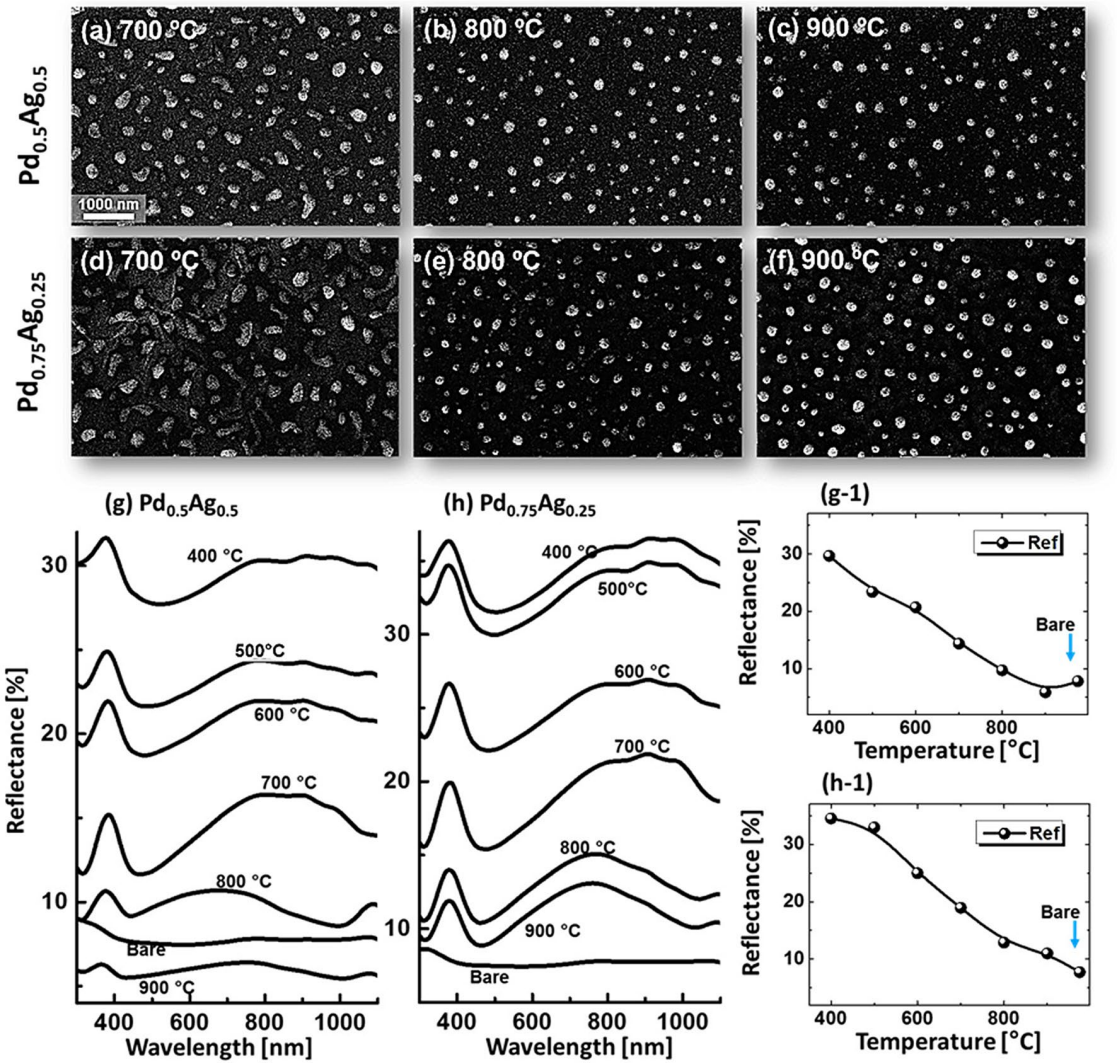
SEM images of Pd-Ag alloy NPs fabricated between 700 and 900 °C with the compositions Pd_0.5_Ag_0.5_ in (a)–(c) and Pd_0.75_Ag_0.25_ in (d)–(f). (g)–(h) Reflectance spectra of the alloy NPs with distinct composition as labeled. (g-1)–(h-1) Average reflectance of corresponding sets in (g) and (h) with respect to the temperature.

Figures 3 and 4 show the evolution of Pd–Ag alloy NPs with the Pd_0.5_Ag_0.5_ and Pd_0.75_Ag_0.25_ with a total thickness of 15 nm annealed at identical environment between 400 and 900 °C for 120 s. The formation of alloy NPs under an identical growth condition can be distinctive due to the composition of Pd and Ag in bilayer films. As discussed, annealing induced transformation of the bilayer thin film into isolated alloy NPs exhibiting various steps: voids and pinholes perforation, voids expansion and coalescence, cluster breakdown and isolated NPs evolution guided by the enhanced surface diffusion and interface inter-mixing at elevated temperature. In general, similar dewetting sequence was demonstrated by all three bilayers compositions. However, the dewetting behavior was altered at relatively low temperatures. For example, the void growth stage was almost completed up to 600 °C for the Pd_0.25_Ag_0.75_ whereas it was still growing up to 600 °C for the Pd_0.5_Ag_0.5_ and up to 700 °C for the Pd_0.75_Ag_0.25_. At the same time, the number of pinholes were reduced, whereas the overlaying grain size was increased in the subsequent sets. As the composition of low diffusivity material (Pd) increased, the overall diffusion can be slower. On the other hand, the formation of Ag grains can be enhanced by the enhanced agglomeration of Ag atoms. The size of overgrown grains was ~90 nm in height and ~400 nm in diameter. After the fragmentation of connected nanoclusters above 700 °C, the well-structured (dome shaped) alloy NPs were formed, which was commonly observed for all three compositions. By comparing the dewetting behavior of three different bilayers compositions, bilayers with the higher Pd composition showed lower diffusivity and vice versa. On the other hand, the size of alloy NPs became larger with the higher Pd thickness, which can be attributed to the reduced Ag amount and sublimation loss. In addition, the NP height and surface coverage were closely monitored by the cross-sectional line profiles, Rq and SAR. Generally, the average height as denoted by the cross-sectional line profiles and Rq, was increased with the temperature. However, for 1:1 composition at higher temperature (900 °C), slight decrease in Rq and average height was observed, which can be to the Ag sublimation. Similarly, the SAR was also amplified along with the surface morphology development. The EDS spectra showed the gradual attenuation of Ag peak count, whereas almost similar Pd peak count was observed within the temperature range. Furthermore, SEM images are shown in Figure 5(a)–(f) and small-scale AFM side-views along with line profiles are shown in Figures S5–S8. For the alloy NPs fabricated with the Pd_0.5_Ag_0.5_ and Pd_0.75_Ag_0.25_ compositions, the reflectance characteristics were slightly varied as compared to the previous set. For low temperature samples (<700 °C), the reflectance response was almost similar for all three compositions. However, the UV peak (~380 nm), visible region absorption band and shoulder in NIR region became more prominent or intense even at high temperature, which can be attributed to the higher composition of Pd. Furthermore, a clear trend of blue shift of the NIR peak was observed above 700 °C, which can be attributed to the reduced size of alloy NPs along with temperature. The initial thickness was reduced for Ag whereas increased for Pd in the succeeding compositions, therefore, the overall sublimation loss can be minimized. This results in the formation of intense reflectance peaks and the NIR peaks position was slightly red-shifted in succeeding compositions. The average reflectance showed the gradually decreasing trend along with the decreased average surface coverage of alloy NPs in all cases. Further, the Raman characteristic showed similar behavior of peak intensity and shift as compared to the previous set as presented in Figures S6 and S8.

**Figure 6.**
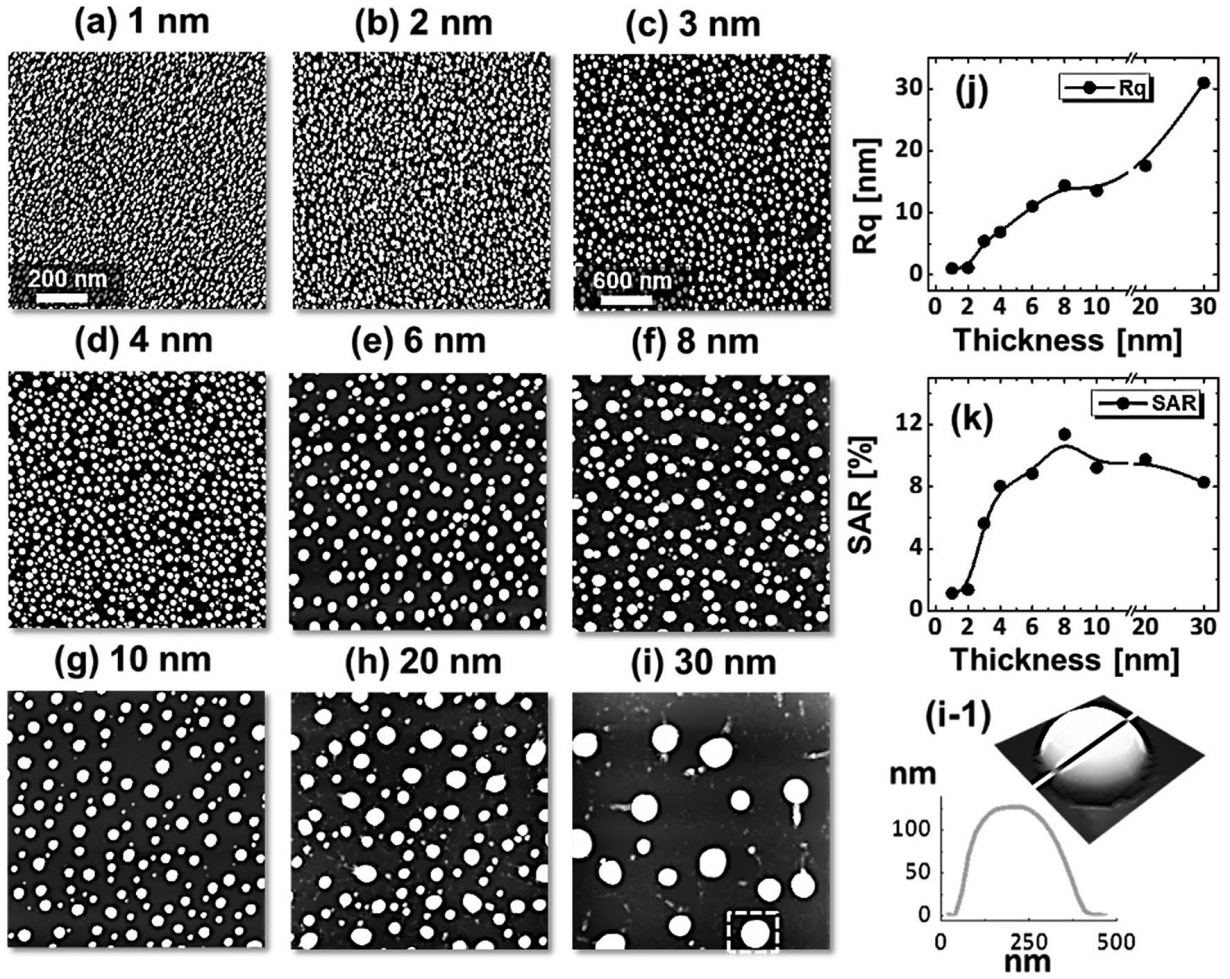
Evolution of dome-shaped Pd_0.25_Ag_0.75_ NPs on sapphire (0 0 0 1), with a thickness between 1 and 30 nm, annealed at 850 °C for 120 s. AFM top-views are 1 × 1 μm^2^ in (a), (b) and 3 × 3 μm^2^ in (c)–(i). (i-1) AFM side-view and line profile of a typical dome-shaped alloy NPs. (j)–(k) Rq and SAR with respect to thickness.

**Figure 7.**
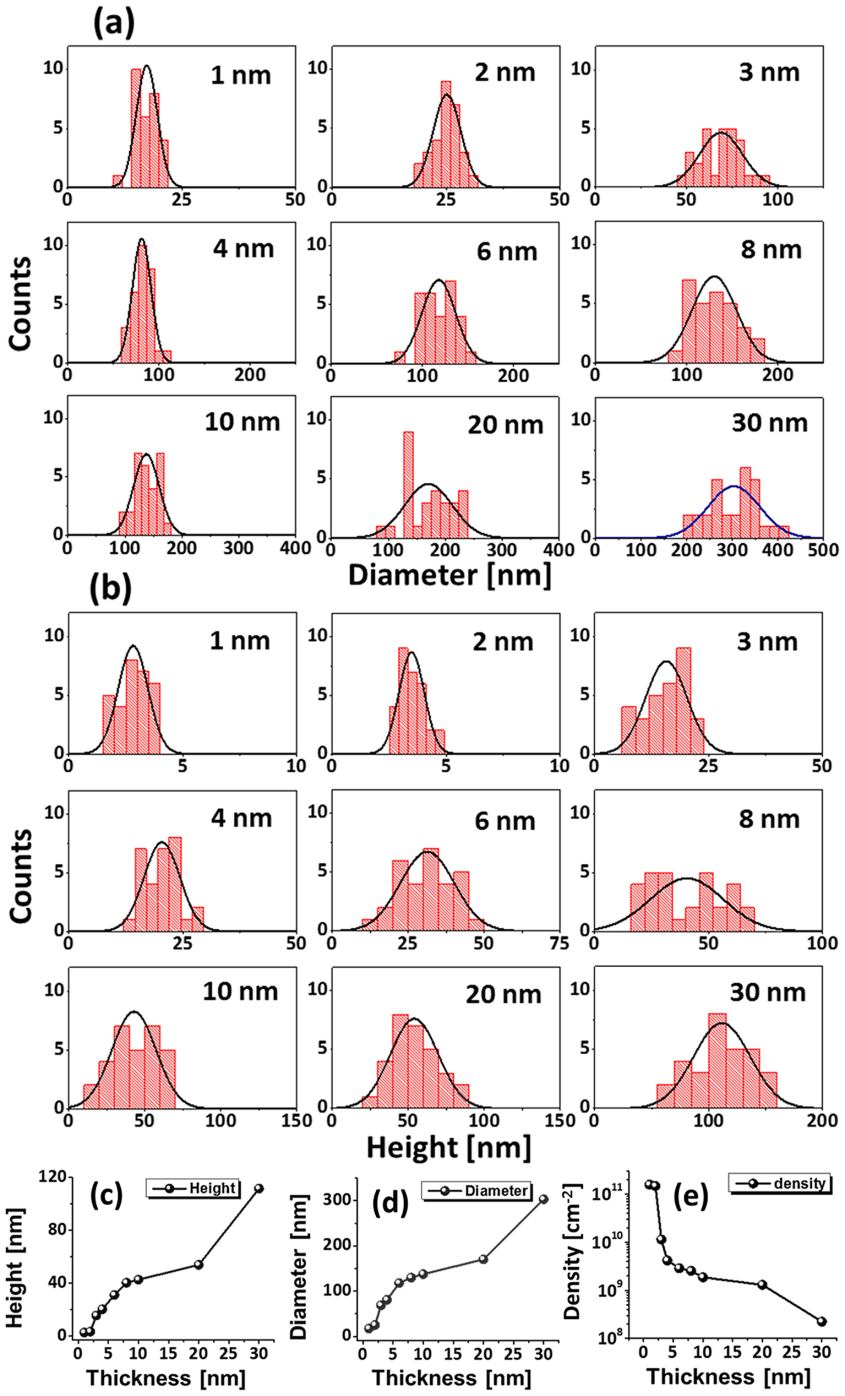
(a) Diameter and (b) height distribution histogram of alloy NPs shown in Figure 6. (c)–(e) Summary plots of average, height, diameter, density with ~±5% error.

**Figure 8.**
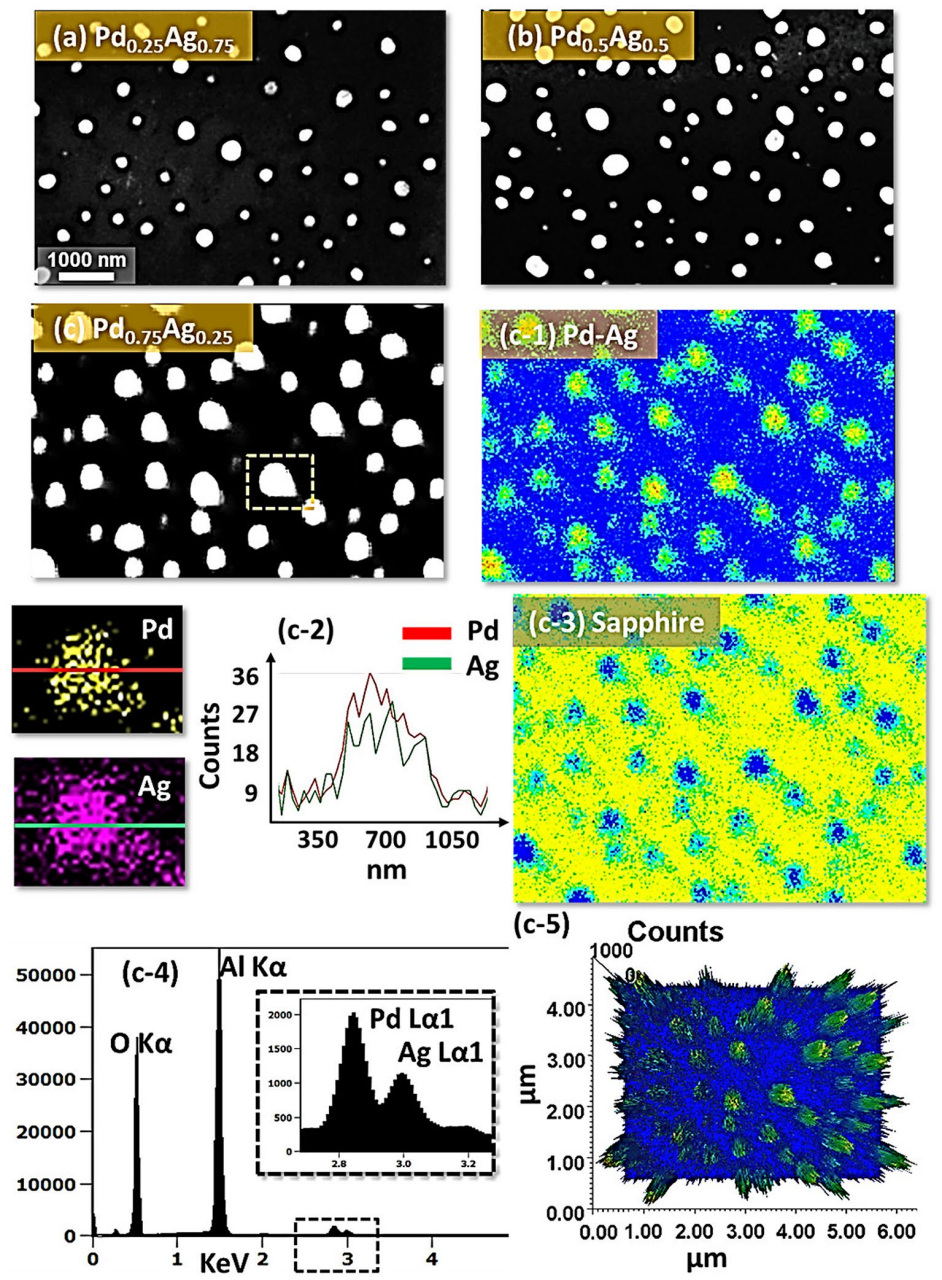
Spherical alloy NPs fabricated at 850 °C for 120 s with 30 nm total thickness for distinct bilayers compositions. SEM images of alloy NPs for (a) Pd_0.25_Ag_0.75_, (b) Pd_0.5_Ag_0.5_, and (c) Pd_0.75_Ag_0.25_ composition. (c-1) Pd-Ag phase map of the alloy NPs in (c). (c-2) Separate Pd and Ag phase maps and EDS line profiles of a selected NP in (c). (c-3) Substrate (Al and O) elemental phase map. (c-4) EDS spectra of the sample showing O, Al, Pd, and Ag peaks. (c-5) 3D top-view of Pd-Ag combined phase map.

Figure 6 shows the fabrication of tiny to large spherical Pd–Ag alloy NPs by controlling the bilayer thickness from 1 to 30 nm at constant temperature and duration with the Pd_0.25_Ag_0.75_ composition. The evolution of alloy NPs is closely monitored in terms of size and density along with the incremental bilayer thickness. As shown in the temperature variation sets, above 850 °C, the alloy NPs fashioned semi-spherical/dome shape attaining significant surface and interface diffusion of bilayers. The initial film thickness has significant impact on the dewetting process of thin film. As discussed earlier, the initial film thickness and void growth rate are inversely proportional, that means the thicker films require higher temperature to achieve agglomeration [40,41]. Generally, once formed NPs tends to absorb diffusing atoms as well as smaller NPs and grow larger in order to gain the equilibrium configuration. With added film thickness, the number of atoms also increased significantly and the growth of NPs can be enhanced [42,43]. Acquiring significant surface diffusion, the growth of alloy NPs can be mainly driven by self-assembly of the NPs toward thermal stability by reducing overall system energy. On the other hand, the thinner films are expected to rupture easily and form small and dense NPs due to the formation of numerous nucleation sites, whereas for higher thickness, the gradual growth of voids can occur rather than forming new void which results in the formation of larger NPs with wider spacing and lower density [44,45]. Therefore, the size, inter-particle spacing and density of alloy NPs could be precisely controlled with the variation of bilayers thickness. As shown in the Figure 6(a), initially, tiny and highly compact alloy NPs were fabricated with the 1 nm total thickness. With small thickness and high annealing temperature the surface adatoms can be sufficiently diffused and became stable at low energy sites resulting tiny isolated NPs. By controlling the bilayers thickness up to 30 nm, the gradual growth in size and corresponding decline in density were witnessed. The surface profiles are investigated by the Rq and SAR as shown in Figure 6(j). The Rq values were consistently increased with thickness, which indicates the gain in the average surface height due to size enlargement of alloy NPs. In case of SAR, it was increased up to 8 nm and gradually decreased for higher DA because of the reduced density and formation of large NPs. In addition, the alloy NPs’ dimensions were investigated by height and diameter histograms, average height, diameter, and density plots as displayed in Figure 7. The right shift of peak of Gaussian distribution curves in height and diameter histograms depict the average size increment. The average height, diameter and density of NPs with 1 nm total thickness were around 3 nm, 17 nm and 15.68 × 10^10^ cm^−2^, respectively, whereas that obtained with 30 nm were around 112 nm, 303 nm, and 2.4 × 10^8^ cm^−2^. In addition, the evolution of Pd–Ag alloy NPs based on distinct bilayer compositions, that is Pd_0.5_Ag_0.5_ and Pd_0.75_Ag_0.25_, were studied within identical deposition range and annealing environment. In which, similar growth trend was observed with the variation in dimension of resulting NPs. The large size alloy NPs for distinct composition are shown in Figure 8 with the detail elemental characterization of 30 nm sample with Pd_0.75_Ag_0.25_ composition. The elemental distribution is presented by EDS phase maps that matched well with SEM image of alloy NPs. Both Pd and Au elements co-exist in alloy NPs as revealed by combined and separated phase maps of Pd and Ag. For a typical NPs selected in the SEM image, the separate Pd and Ag phase mapping are shown along with the EDS line profile in Figure 8(c-2).

**Figure 9.**
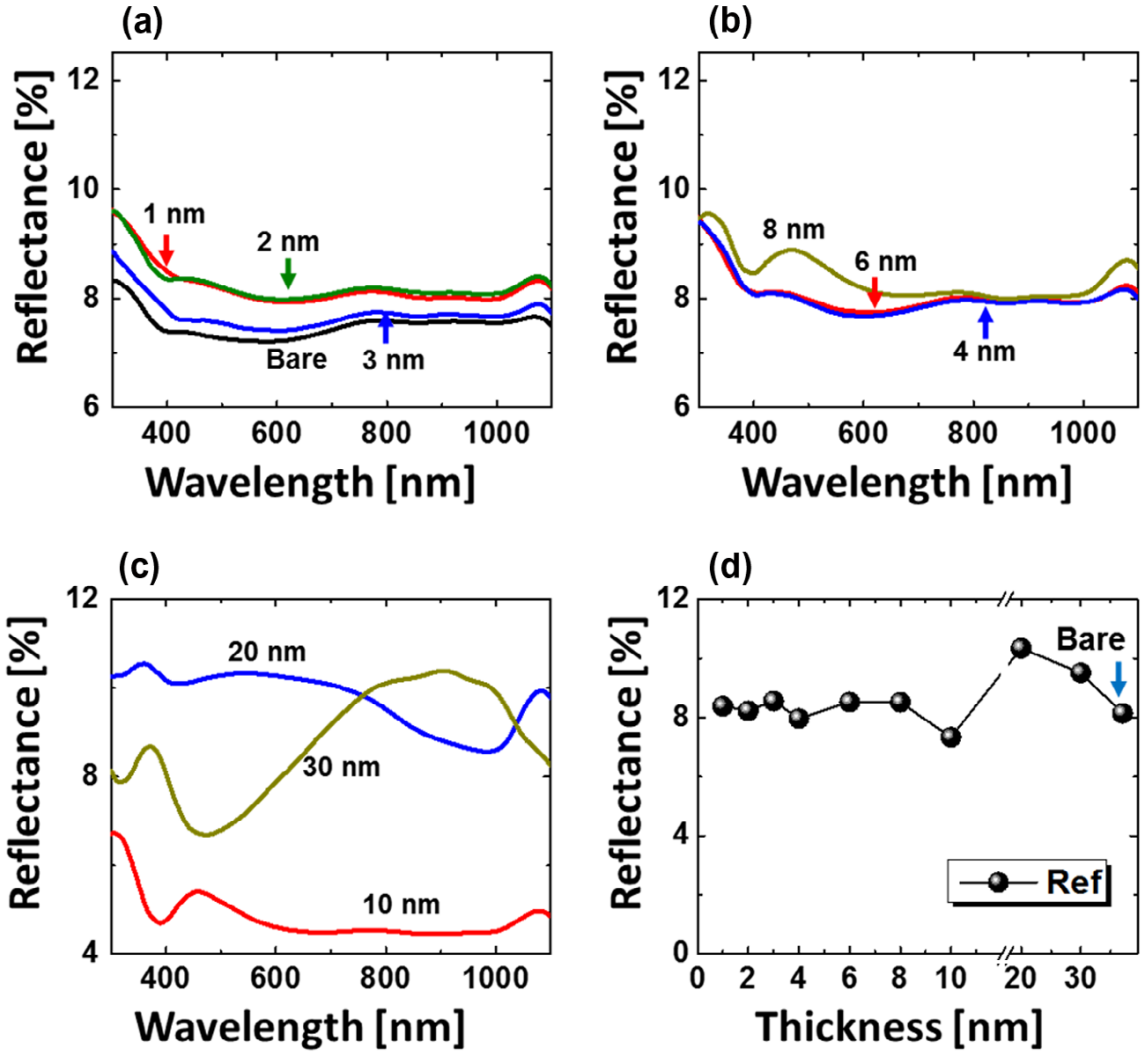
Reflectance spectra of the alloy NPs fabricated at 850 °C for 120 s with composition Pd_0.25_Ag_0.75_ and total thickness variation (a) 1–3 nm, (b) 4–8 nm, and (c) 10–30 nm. (d) Average reflectance with respect to thickness.

Figure 9 presents optical characterization results of dome-shaped Pd_0.25_Ag_0.75_ NPs. For other compositions, the reflectance spectra and detail analysis are provided in supplementary information. As shown in Figure 9(a), the reflectance characteristic was almost similar up to 10 nm with a minor deviation in average reflectance. The surface consists highly dense alloy NPs up to 8 nm, which may result in the broadband absorption of incident light. The absorption enhancement can be very large for the alloy NPs that are much smaller than the wavelength of light. As seen from the size characterization, the NPs size was less than 300 nm, which is less than the wavelength of incident light. Along with the formation of large and widely spaced alloy NPs, the light scattering can be enhanced due to the large scattering cross-section. Therefore, the average reflectance was slightly increased up to 20 nm thickness. On the other hand, the reflectance spectra showed small bump in the visible region for 10 nm thickness and gradually right shifted for 20 and 30 nm, respectively, as shown in Figure 9(c). For large and widely spaced alloy NPs with 20–30 nm total thickness, absorption in visible region was largely enhanced, which can be the consequence of the surface plasmon resonance induced by the widely-spaced large alloy NPs. Meanwhile, the peak in the visible region was red-shifted along with size of alloy NPs between 10 and 30 nm. Similarly, for other compositions, there was no discernible peak or valley formation for highly dense small NPs up to 3 nm. However, with medium size alloy NPs between 4 and 8 nm, a dip in UV (~380 nm) and a wide trough in UV (~380 nm) NIR region (~700–1000 nm) were detected. Large alloy NPs with 20 and 30 nm thickness exhibited sharp absorption of visible wavelength, whereas enhanced reflection of UV and NIR wavelength. The Raman spectra showed the inverse relation of peak intensity with the surface coverage of alloy NPs along with minor left shift as shown in Figures S11, S15, and S18.

## 4. Conclusions

In summary, the Pd–Ag alloy NPs were fabricated on sapphire (0 0 0 1) using sputtering deposition technique and subsequent annealing. The dewetting of bilayer films was systematically controlled by the annealing temperature, thickness, and composition of bilayers, which correspondingly resulted in the distinct shape, size, and density of alloy NPs. The increased temperature between 400 and 900 °C with the fixed thickness demonstrated various stages of alloy NP growth such as: void nucleation, followed by expansion and finally isolated NPs formation and shape transformation toward semi-spherical configuration. At temperature above 500 °C, Ag content in alloy NPs was reduced as compared to the initial deposited due to the sublimation, which also reduce the size of alloy NPs. In addition, the tiny and dense NPs to the enlarged with wide spacing were fabricated based on the incremental variation of bilayer thickness between 1 and 30 nm. Furthermore, the reflectance spectra revealed that the average reflectance has direct relationship with average surface coverage and the absorption enhancement and shift were dependent with size and elemental composition of alloy NPs. The Raman spectra exhibited reduced intensity with the high surface coverage. The finding from this work can be useful in the design and synthesis of efficient plasmonic and catalytic applications using simple and reproducible solid-state dewetting approach.

## Disclosure statement

No potential conflict of interest was reported by the authors.

## Funding

This work was financially supported by the National Research Foundation of Korea [grant numbers 2011-0030079; 2016R1A1A1A05005009], and in part by the research grant of Kwangwoon University in 2017.

## Supplemental data

Supplemental data for this article can be accessed at https://doi.org/10.1080/14686996.2018.1435944.

## Supplementary Material

Supplementary_Materials_for_Publication.docx
