# Supplementary material for: Morphological and optical properties of PdxAg1-x alloy nanoparticles
Source: Sci Technol Adv Mater. 2018 Feb 22;19(1):160–73. doi: 10.1080/14686996.2018.1435944 (PMC5827785; doi:10.1080/14686996.2018.1435944)
Supplement: Supplementary_Materials_for_Publication.docx [file TSTA_A_1435944_SM8027.docx]

**Supplementary Materials
for Publication**

**Morphological and Optical Properties of Pd_x_Ag_1-x_ Alloy Nanoparticles**

**Sundar Kunwar^1^, Puran Pandey^1^, Mao Sui^1^, Sushil Bastola^1^ and Jihoon Lee^1,2*^**

^1^College of Electronics and Information, Kwangwoon University, Nowon-gu Seoul 01897, South Korea ^2^Institute of Nanoscale Science and Engineering, University of Arkansas, Fayetteville AR 72701, USA.

E-mail: jihoonleenano@gmail.com

**
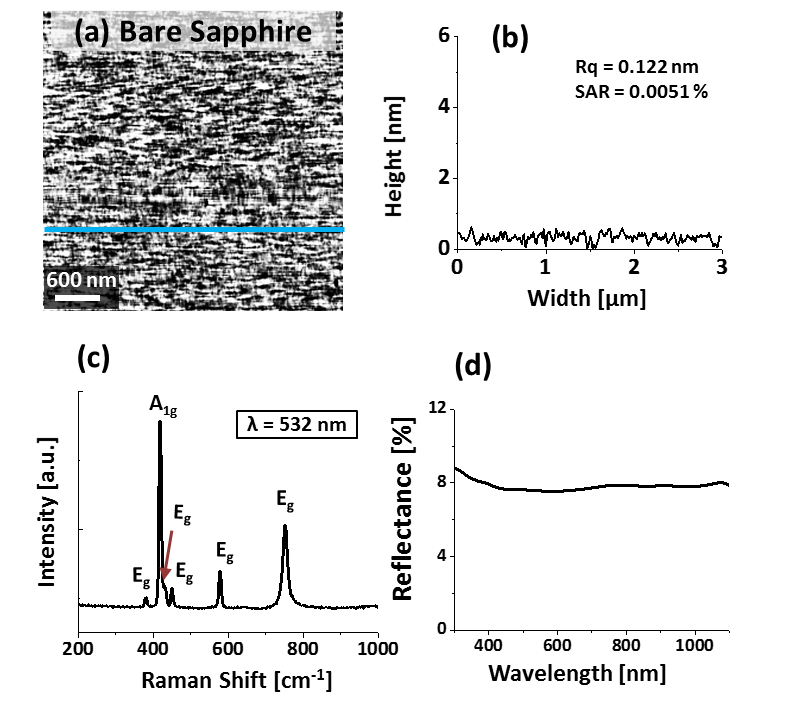
**

**Figure S1:** (a) Surface morphology of bare sapphire (0001) as obtained by the AFM scanning. The AFM top-view is 3 × 3 µm^2^. (b) Corresponding line profile depicts surface profile of bare sapphire. (c) Raman spectra of the bare sapphire (0001) excited by 532 nm at 220 mW, which exhibited six natural vibration modes, namely: A_1g_ at ~ 417 cm^-1^ and E_g_ at ~ 379, 429, 448, 577 and 750 cm^-1^. (d) UV-VIS-NIR reflectance spectra of bare sapphire which showed the almost uniform response throughout the wavelength.

**
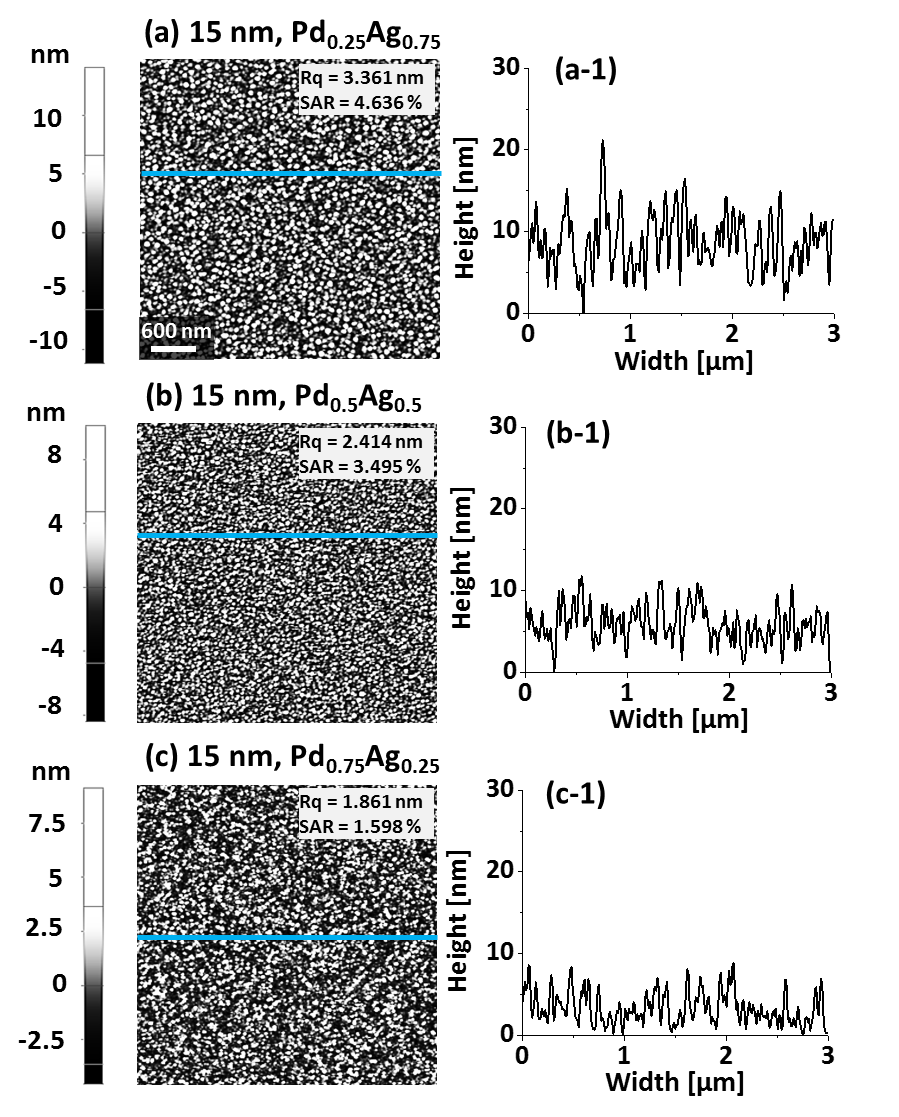
**

**Figure S2:** AFM top-views show the surface morphology of pre-annealed samples after the deposition of Pd and Ag bi-layers with distinct compositions at 15 nm total thickness: (a) Pd_0.25_Ag_0.75_, (b) Pd_0.5_Ag_0.5_ and (c) Pd_0.75_Ag_0.25_. As seen in the AFM images the surface morphology differed for different composition of bilayers. (a-1) – (c-1) Corresponding line profiles of the AMF top-views.

**
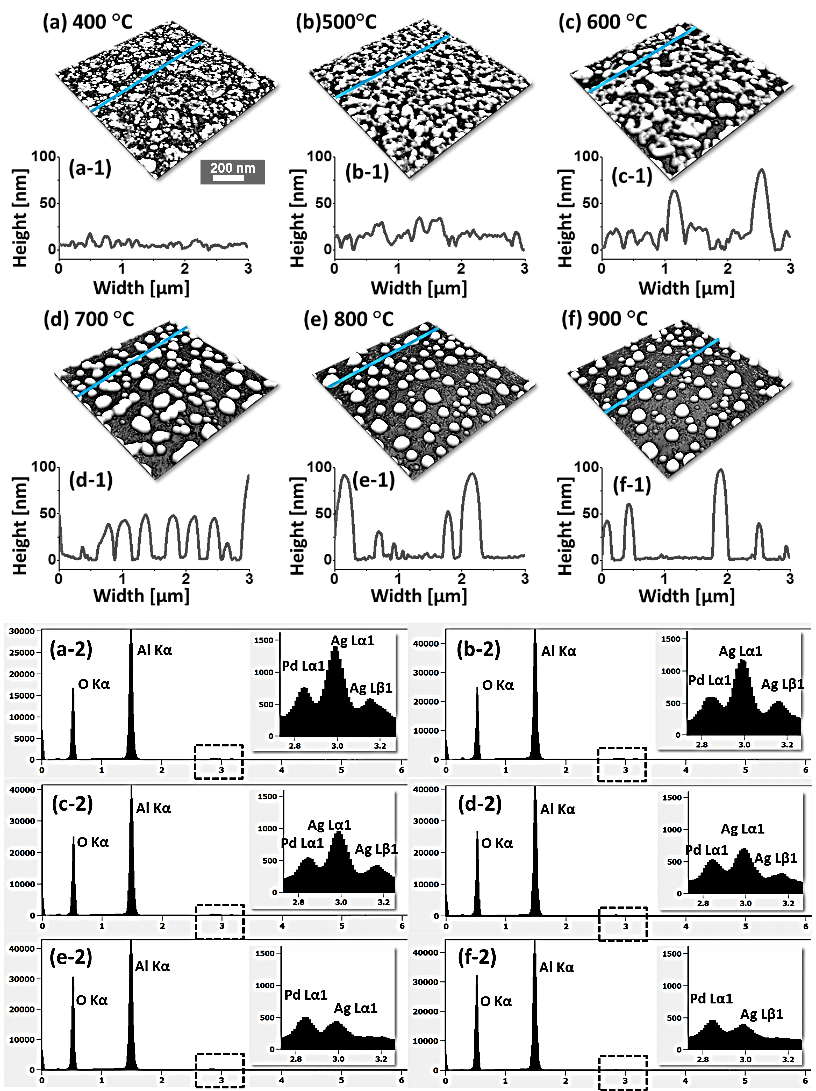
**

**Figure S3:** Effect of annealing temperature (400 – 900 ºC) on the evolution of Pd-Ag alloy NPs on sapphire (0001). The total thickness of 15 nm with Pd_0.25_Ag_0.75_ were annealed for 120 s at each temperature. (a) – (f) AFM side-views of 1 × 1 µm^2^. (a-1) – (f-1) Cross-sectional line profiles. (a-2) – (f-2) EDS spectra show the peaks corresponding to the element of samples. Insets show the peaks of Ag Lα1, Ag Lβ1 and Pd Lα1.

**
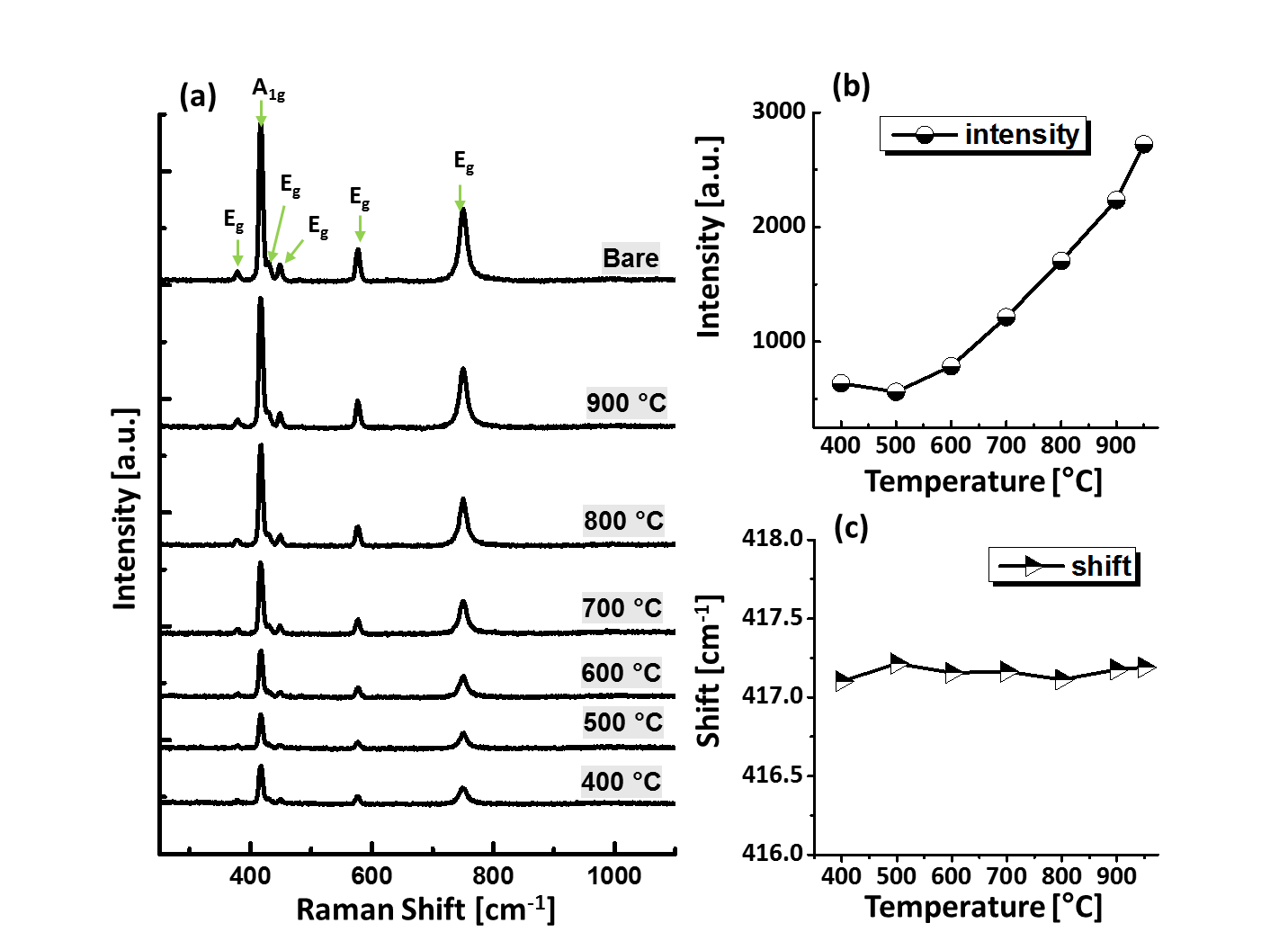
**

**Figure S4:** Raman spectra (200 – 1100 cm^-1^) of samples shown in Fig S1. Six vibration modes namely; E_g_ ( ~ 379 cm^-1^), A_1g_ ( ~ 417 cm^-1^), E_g_ ( ~ 429 cm^-1^), E_g_ ( ~ 448 cm^-1^), E_g_ ( ~ 577 cm^-1^) and E_g_ ( ~ 750 cm^-1^) are measured by 532 nm at 220 mW laser at room temperature. All samples consist of these six vibration modes and the intensity was inversely related to the average surface coverage of the alloy NPs.

**
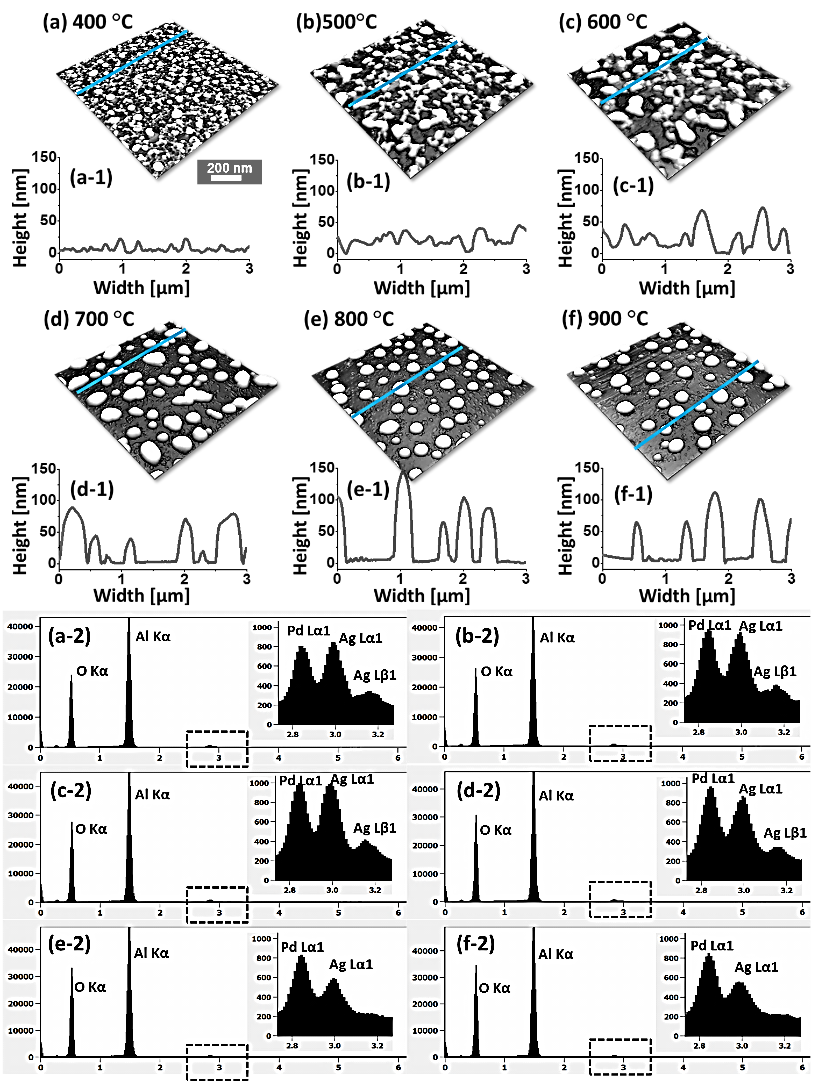
**

**Figure S5:** Pd-Ag alloy NPs on sapphire (0001) annealed at identical conditions (between 400 and 900 °C for 120 s). The total film thickness of 15 nm consists of 7.5 nm Pd and Ag (Pd_0.5_Ag_0.5_). (a) – (f) AFM side-views of 1 × 1 µm^2^. (a-1) – (f-1) Cross-sectional line profiles of AFM images in (a) – (f). (a-2) – (f-2) EDS spectra show the peaks corresponding to the elements in each sample. Insets show the peaks related to Pd and Ag.

**
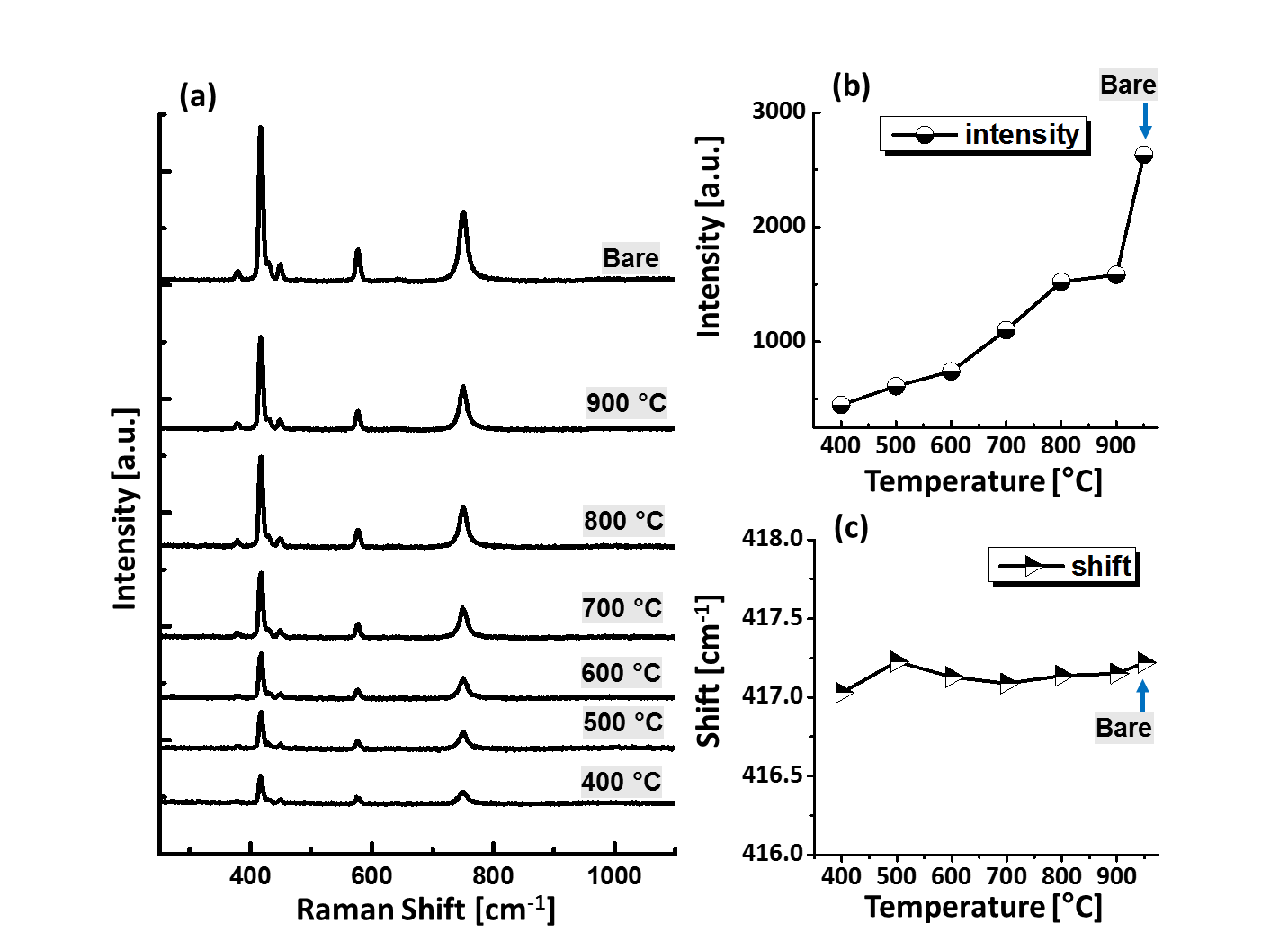
**

**Figure S6:** Raman spectra of the samples alloy NPs on sapphire shown in Fig S3. (a) The six vibration modes of sapphire commonly observed in each sample. (b) – (c) Summary plots of peak intensity and shift based on A_1g_ vibration mode.

**
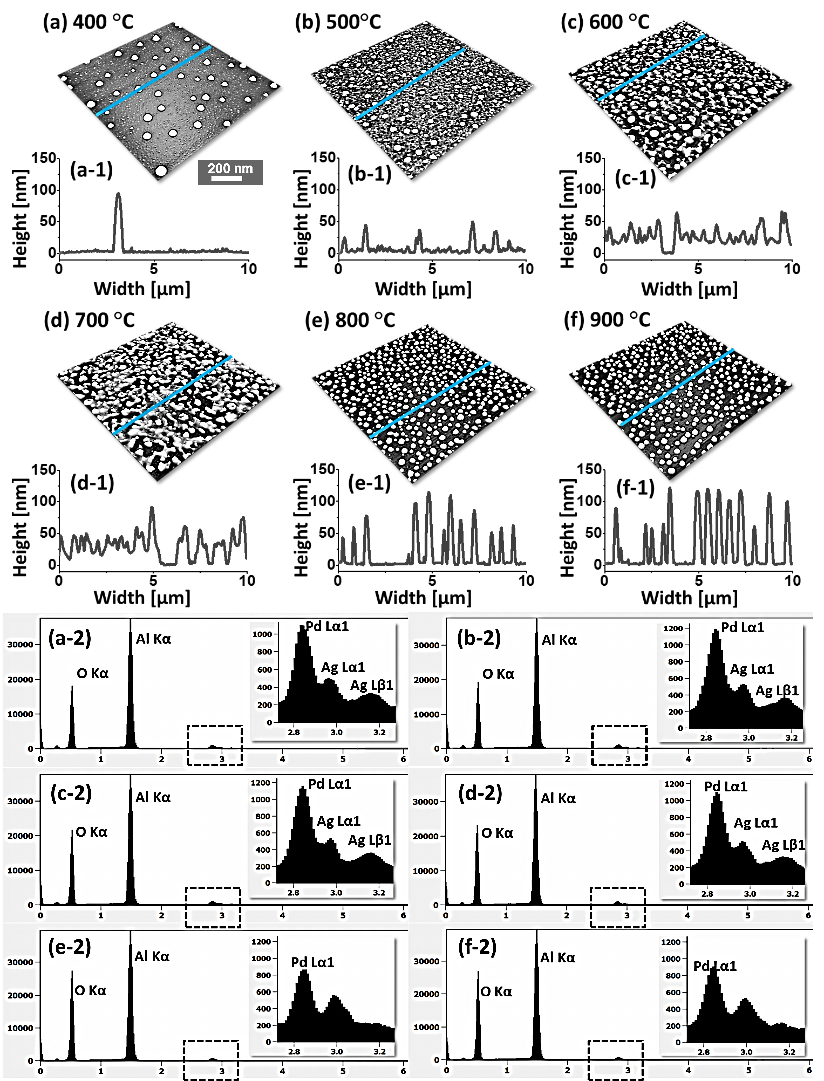
**

**Figure S7:** Evolution alloy NPs on sapphire (0001) with composition Pd_0.75_Ag_0.25_, annealed at identical environment between 400 and 900 °C for 120 s. (a) – (f) AFM side-views of 1 × 1 µm^2^. (a-1) – (f-1) Cross-sectional line profiles. (a-2) – (f-2) EDS spectra shows the peaks corresponding to the element of samples. Insets show the peaks related to Pd and Ag.

**
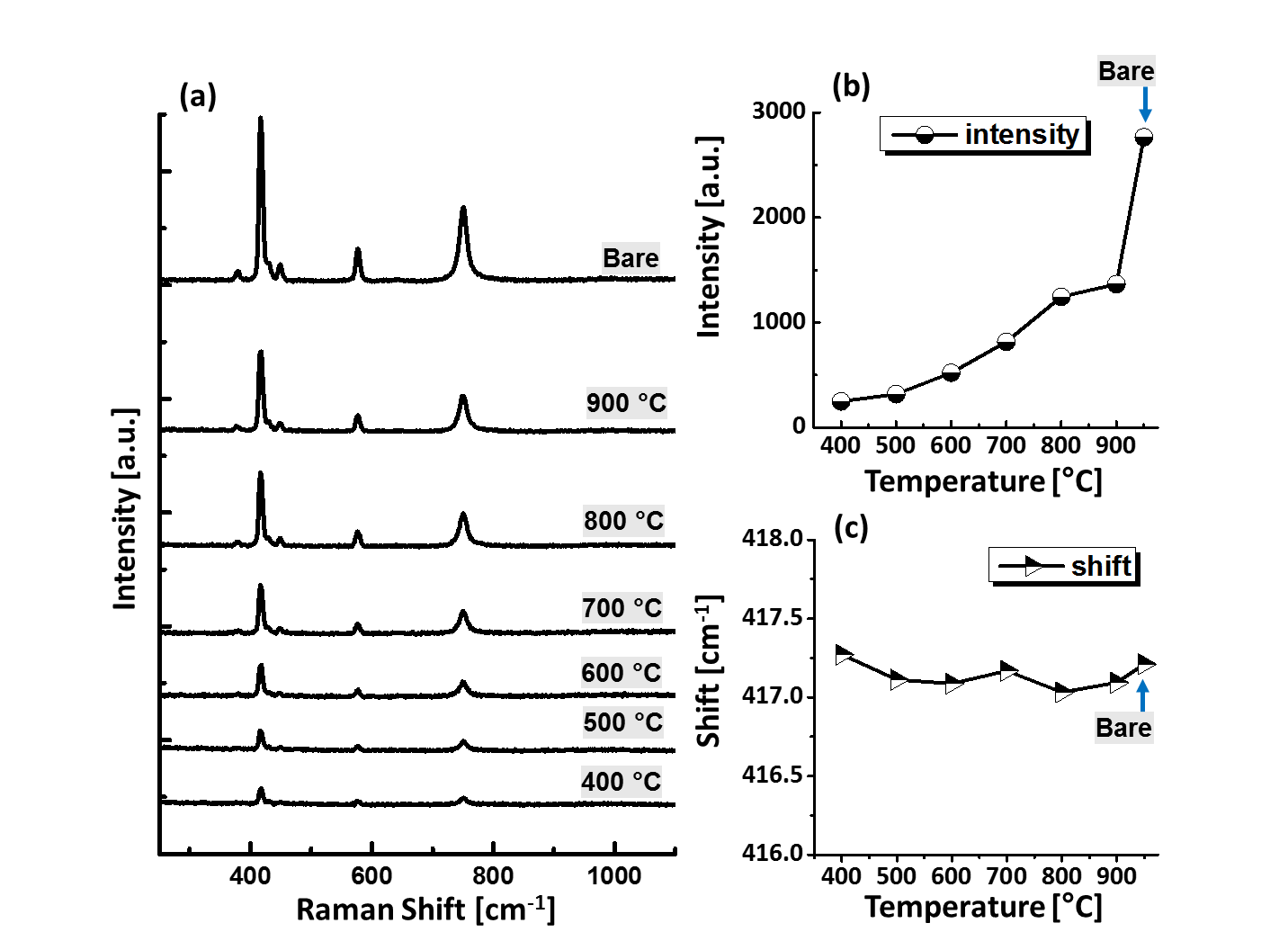
**

**Figure S8:** Raman spectra of the samples shown in Fig S5. (a) Six vibration modes of sapphire are detected in each sample with A_1g_ as a most intense peak which was used to characterize the Raman characteristics. (b) – (c) Summary plot of peak intensity and shift based on A_1g_ vibration mode.

**
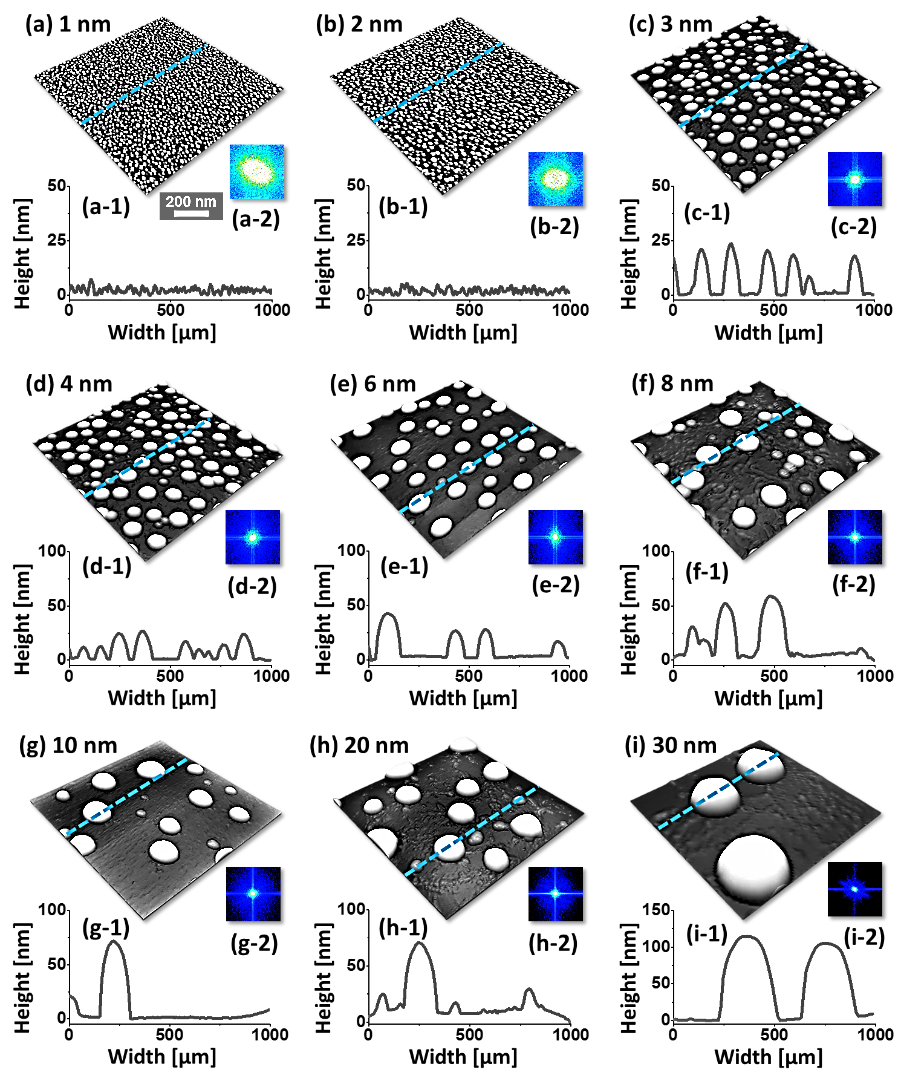
**

**Figure S9:** Formation of tiny to large dome shaped Pd-Ag alloy NPs on sapphire (0001) based on the total thickness variation between 1 and 30 nm with fixed bilayer composition Pd_0.25_Ag_0.75_. The fabrication was performed in identical environment at 850 °C for 120 s. (a) – (i) AFM images of 1 × 1 µm^2^. (a-1) – (i-1) Cross-sectional line profiles of images in (a) – (i). (a-2) – (i-2) 2D Fourier filter transform (FFT) spectra of corresponding AFM side-views.

**
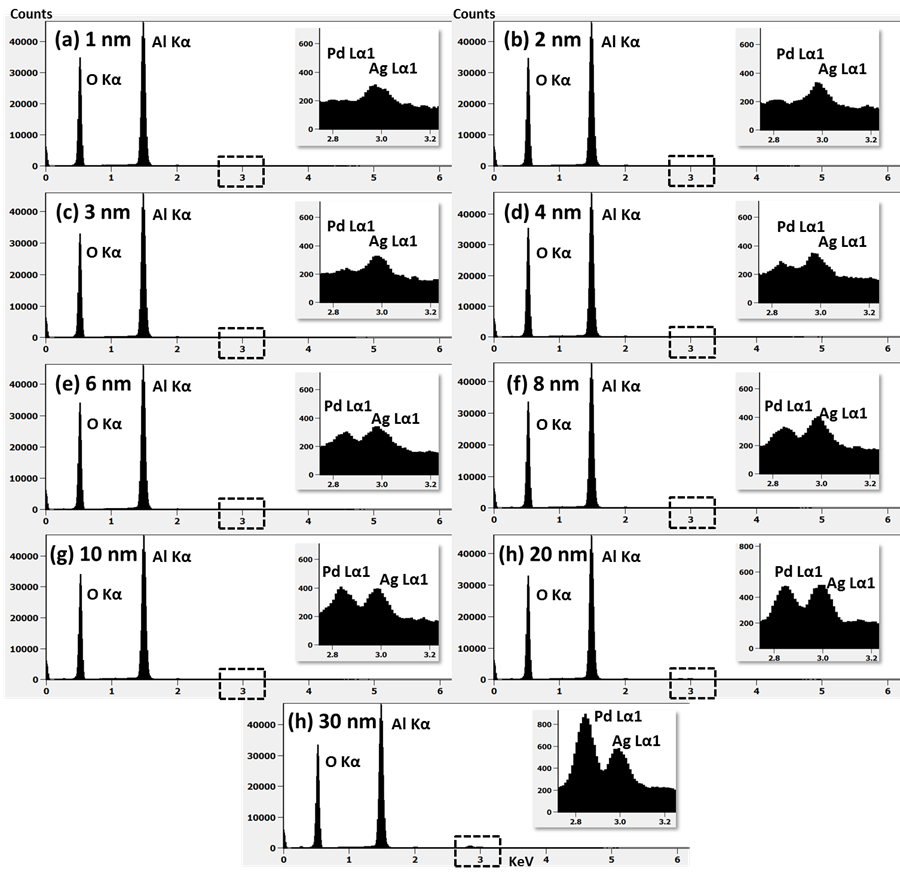
**

**Figure S10:** (a) – (h) EDS spectra within 0 to 6.5 keV show the peaks corresponding to Al, O, Pd and Ag present in the samples shown in Fig. S7. The intensity of peaks denotes the amount of element. Insets highlight the Pd and Ag peaks namely Pd Lα1 at 2.836 keV and Ag Lα1 at 2.981 keV respectively.

**
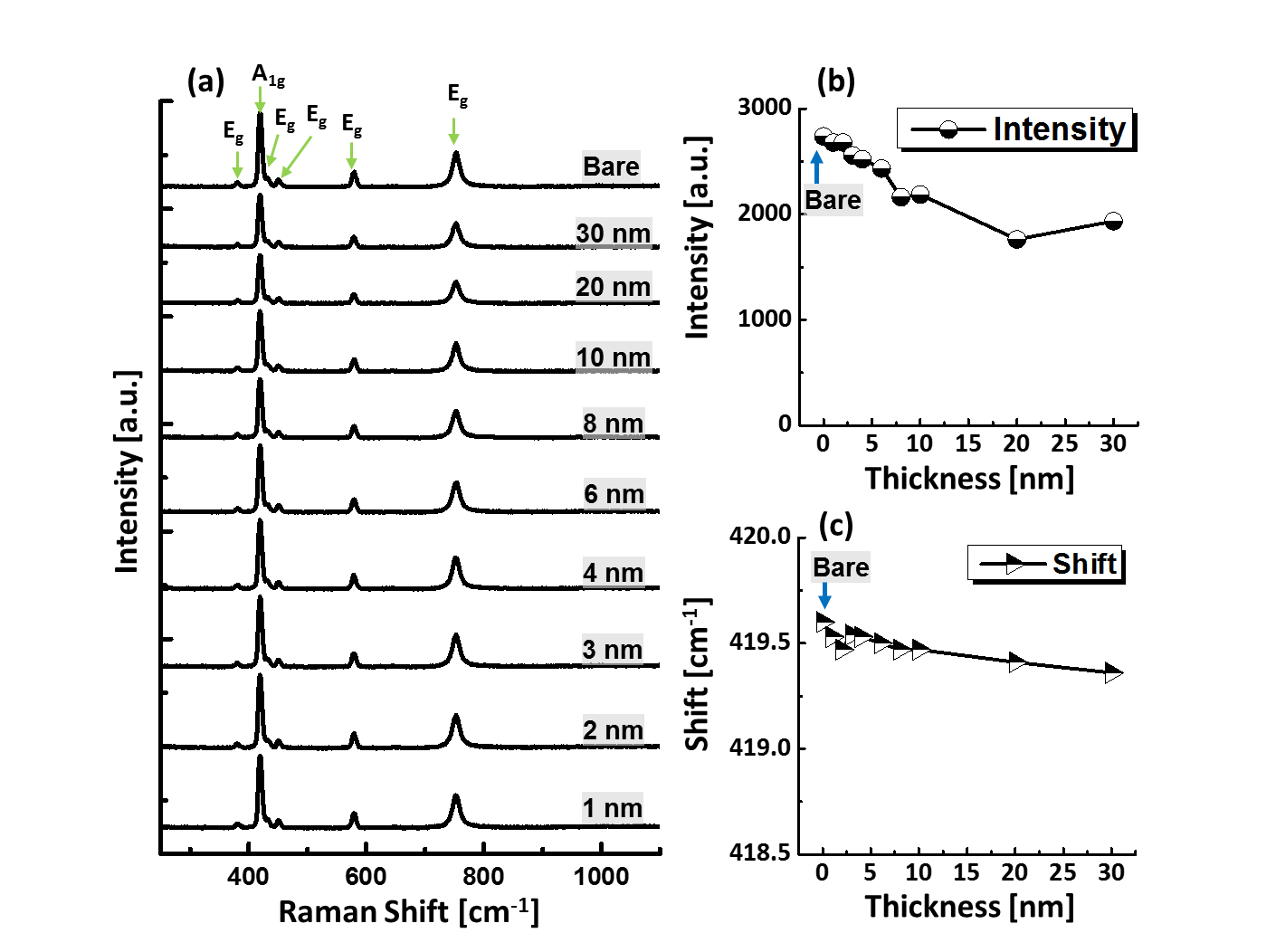
**

**Figure S11: (a)** Full range (200 – 1100 cm^-1^) Raman spectra showing six vibration modes of sapphire (0001) as depicted in each sample shown in Fig. S7. The total thickness was consisted of Pd and Ag bilayer in a ratio Pd_0.25_Ag_0.75_. (b) – (c) Summary plots of peak intensity and shift with respect to thickness.

**
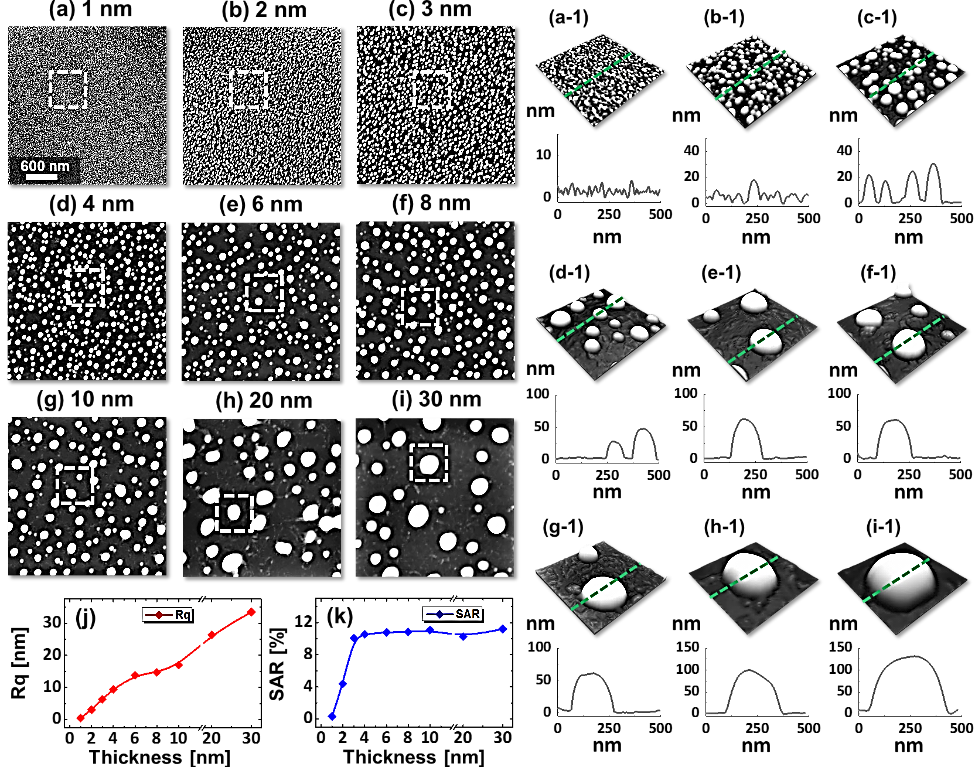
**

**Figure S12:** Tiny to large dome shaped Pd-Ag alloy NP evolution by the variation of thickness from 1 to 30 nm with a fixed composition of Pd_0.5_Ag_0.5_ annealed at 850 ºC for 120 s. (a) – (i) AFM top-views of 3 × 3 µm^2^. (a-1) – (i-1) AFM side-views of selected regions in images (a) – (i) and corresponding line profiles. (j) – (k) Summary of Rq and SAR with respect to thickness.

**
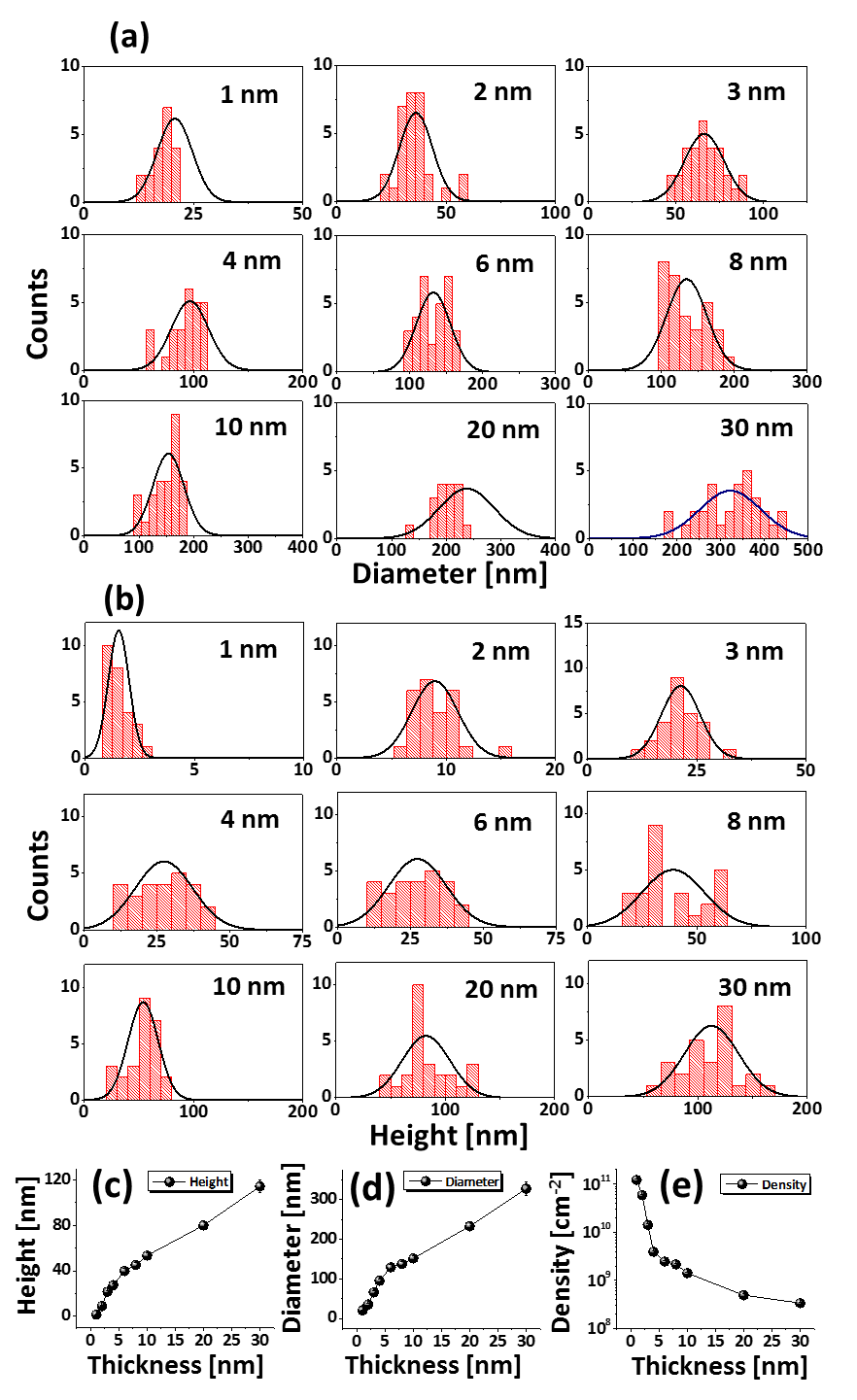
**

**Figure S13:** (a) Diameter and (b) height distribution histogram of alloy NPs shown in Fig. S10. (c) – (e) Summary plots of average, height, diameter, density with ± 5 % error bar.

**
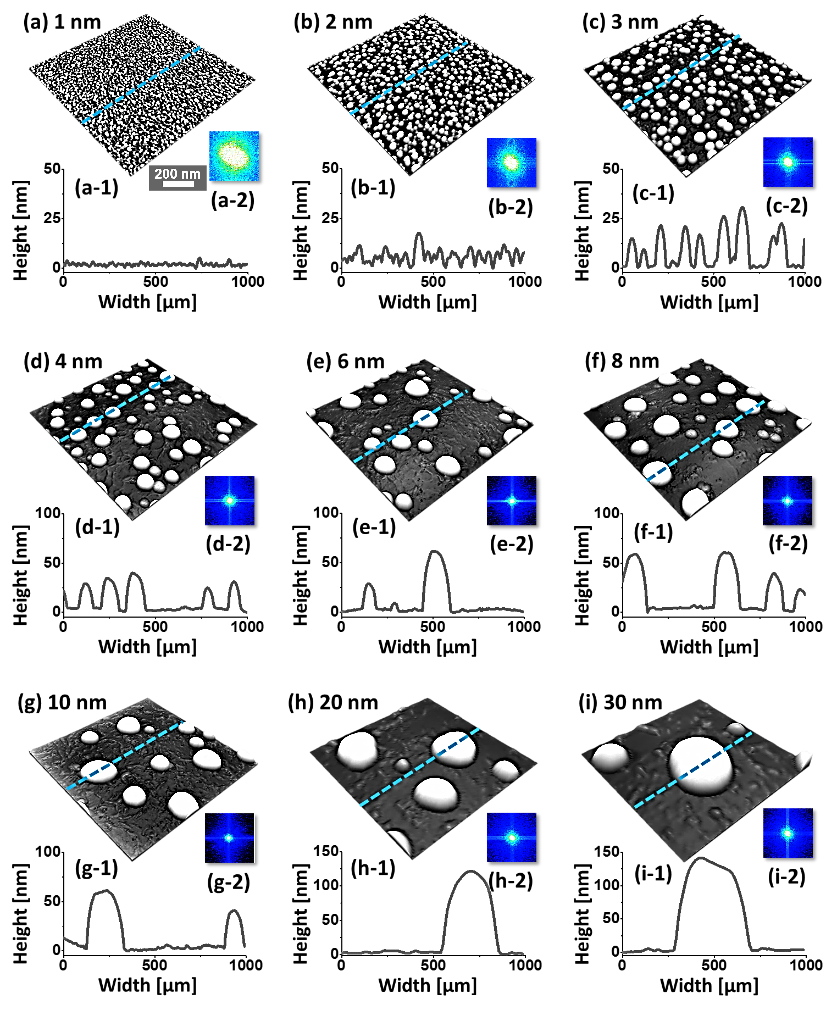
**

**Figure S14:** Evolution of tiny to large dome shaped alloy NPs with fixed composition of bilayer i.e. Pd_0.5_Ag_0.5_ based on the total thickness variation between 1 and 30 nm. The fabrication was performed in an identical environment at 850 °C for 120 s. (a) – (i) AFM images of 1 × 1 µm^2^. (a-1) – (i-1) Cross-sectional line profiles of images in (a) – (i). (a-2) – (i-2) FFT spectra of corresponding AFM side-views.

**
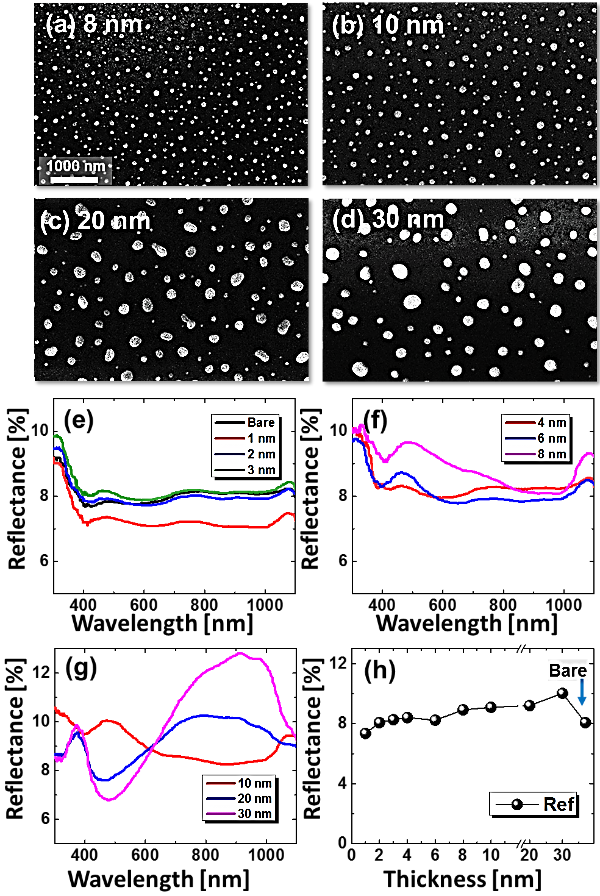
**

**Figure S15:** (a) – (d) SEM images of alloy NPs with the fixed composition Pd_0.5_Ag_0.5_ and total thickness varied between 8 and 30 nm. The annealing was performed at 850 °C for 120 s. (e) – (g) UV-VIS-NIR reflectance spectra of alloy NPs on sapphire (0001). (h) Summary plot of average reflectance with respect to thickness.

**
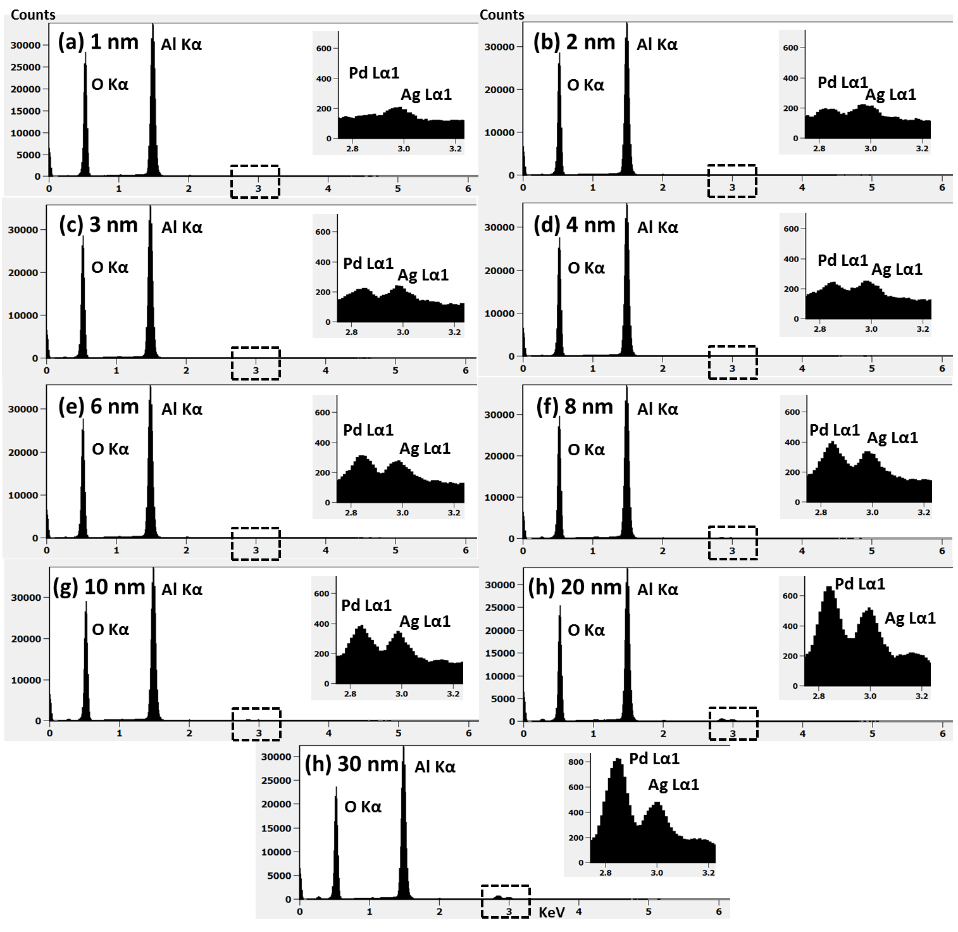
**

**Figure S17:** (a) – (h) EDS spectra within 0 to 6.5 keV show the Al, O, Pd and Ag peaks of the samples with increasing thickness as displayed in Fig. S10 (Pd_0.5_Ag_0.5_). The intensity of the peaks denotes the amount of element. Insets highlights the Pd and Ag peaks namely Pd Lα1 at 2.836 keV and Ag Lα1 at 2.981 keV respectively.

**
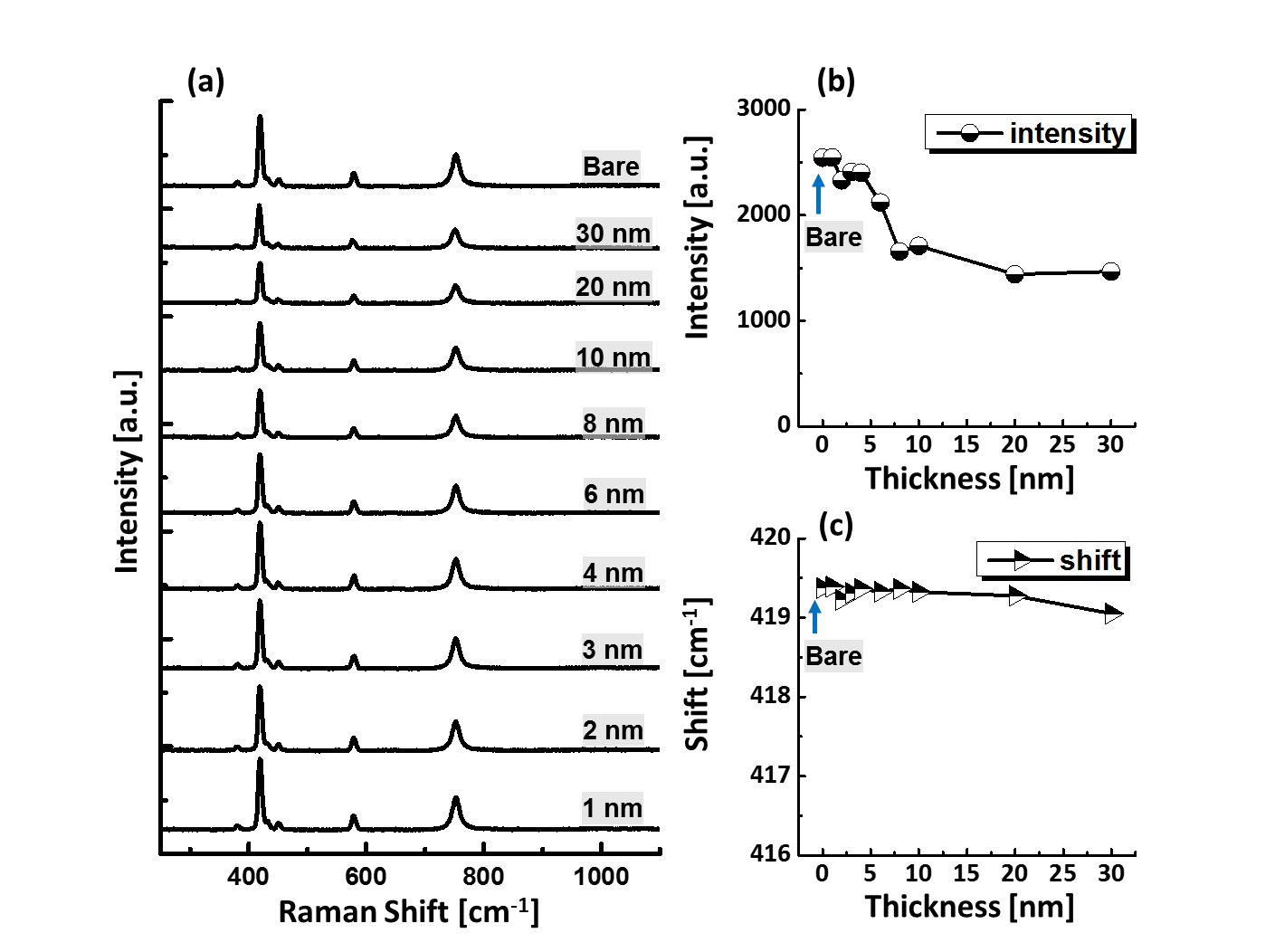
**

**Figure S18:** (a) Raman spectra of samples shown in Fig. S10 (Pd_0.5_Ag_0.5_) with various thickness as labelled. (b) - (c) Summary plots of peak intensity and shift with respect to thickness.

**
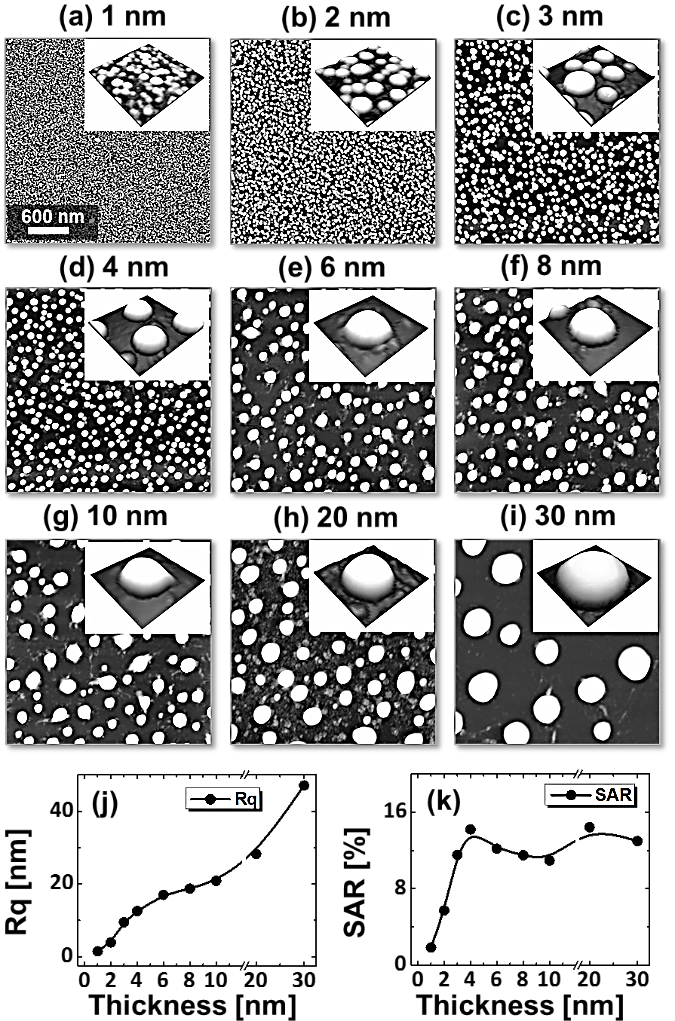
**

**Figure S19:** Size evolution of dome shaped Pd-Ag alloy NPs at 850 ºC for 120 s with fixed composition of Pd and Ag (Pd_0.75_Ag_0.25_) and total thickness between 1 and 30 nm. (a) – (i) AFM top-views of 3 × 3 µm^2^. Insets show the side-views of typical NPs from the corresponding large AFM images. (j) – (k) Summary plots of Rq and SAR with respect to thickness.

**
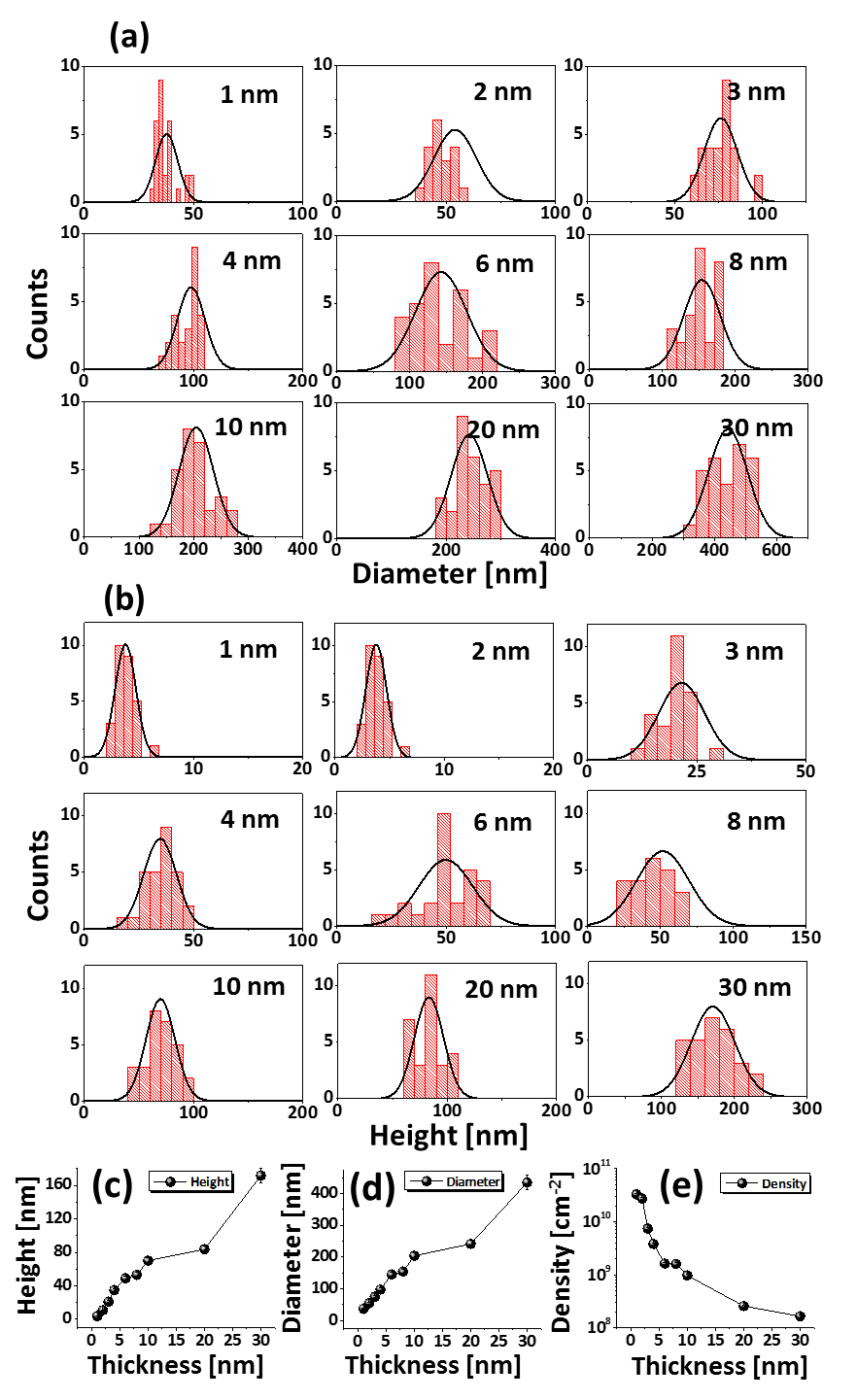
**

**Figure S20:** (a) Diameter and (b) height distribution histogram of alloy NPs (Pd_0.75_Ag_0.25_) shown in Fig. S16. (c) – (e) Summary plots of average, height, diameter, density with ± 5 % error bar.

**
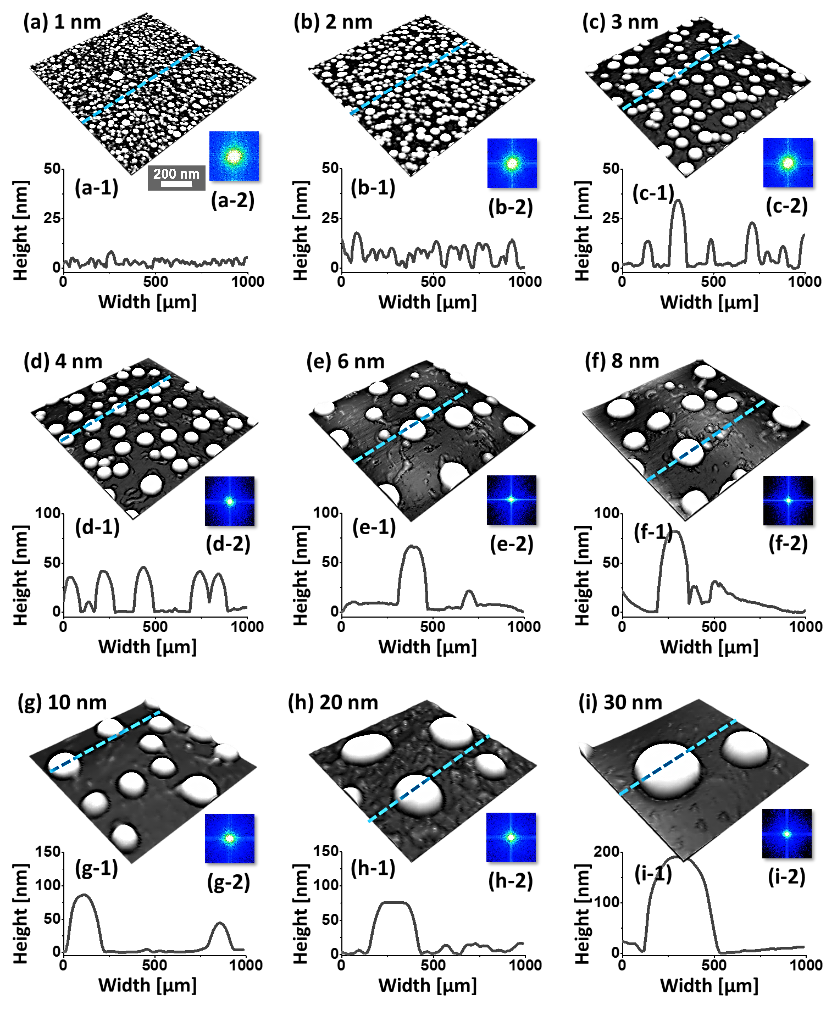
**

**Figure S21:** Evolution of tiny to large dome shaped Pd-Ag alloy NPs with the composition Pd_0.75_Ag_0.25_ based on the total thickness variation between 1 and 30 nm. The fabrication was performed in identical environment at 850 °C for 120 s. (a) – (i) Small scale AFM images of 1 × 1 µm^2^. (a-1) – (i-1) Cross-sectional line profiles of lines in (a) – (i). (a-2) – (i-2) 2D FFT spectra of corresponding AFM images.

**
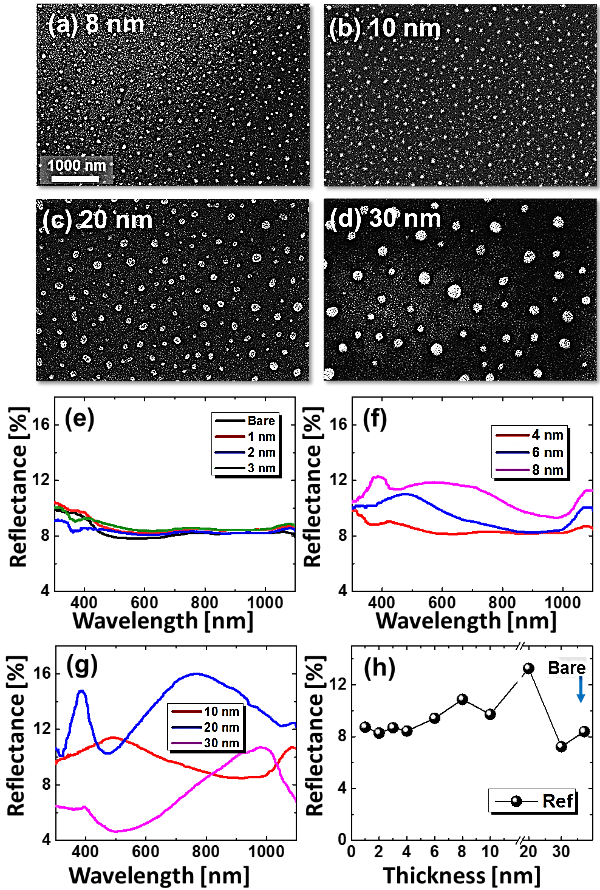
**

**Figure S22:** (a) – (d) SEM images of alloy NPs with the composition ratio Pd_0.75_Ag_0.25_ and total thickness varied between 8 and 30 nm. The annealing was performed at 850 °C for 120 s. (e) – (g) Reflectance spectra of Pd-Ag alloy NPs between 300 and 1100 nm wavelength. (h) Summary plot of average reflectance with respect to thickness.

**
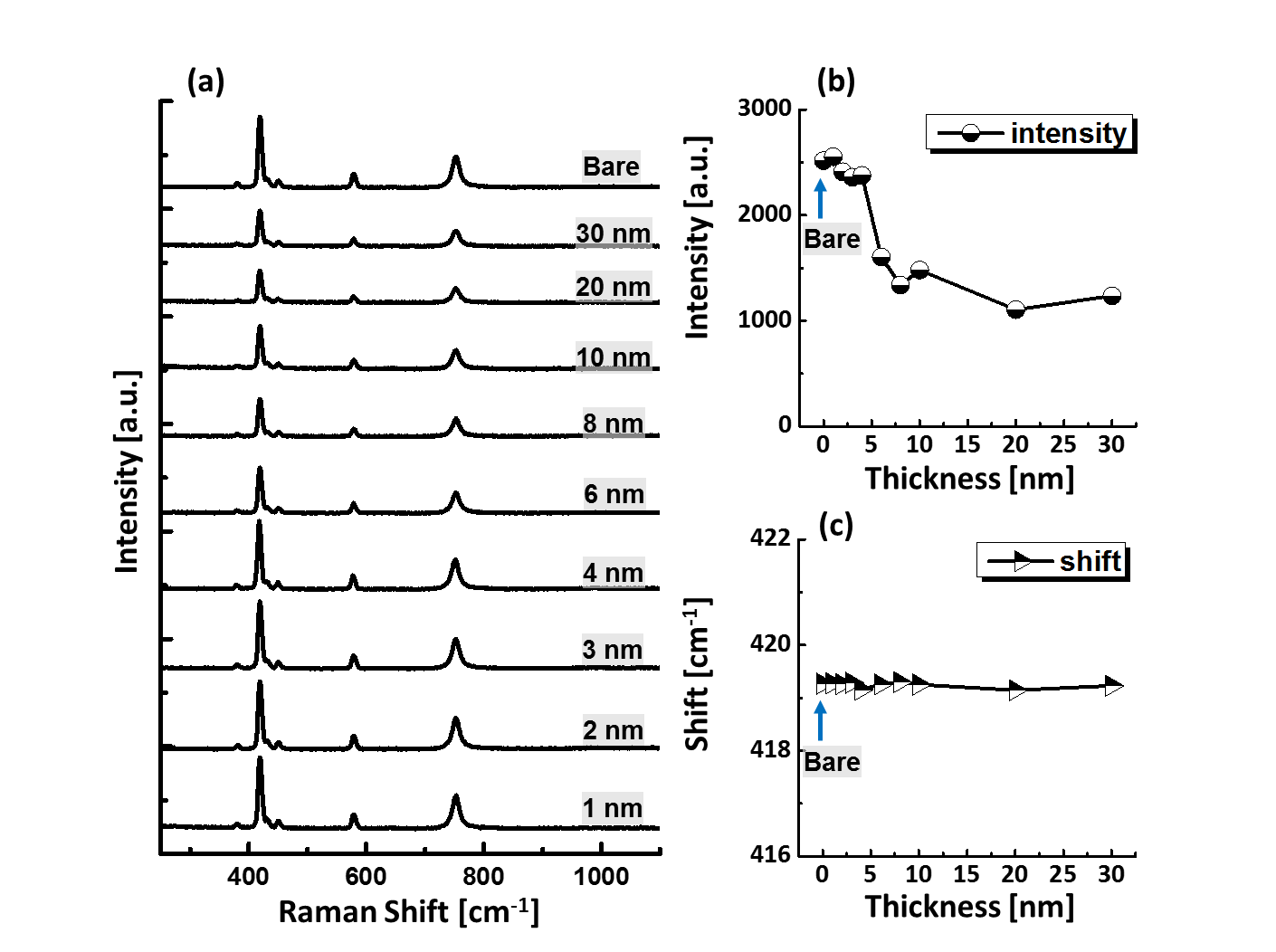
**

**Figure S23:** (a) Raman spectra of Pd-Ag nanostructures on sapphire (0001) presented in Fig S16. (b) - (c) Summary plots of peak intensity and shift with respect to thickness probed by A_1g_ vibration mode.

**
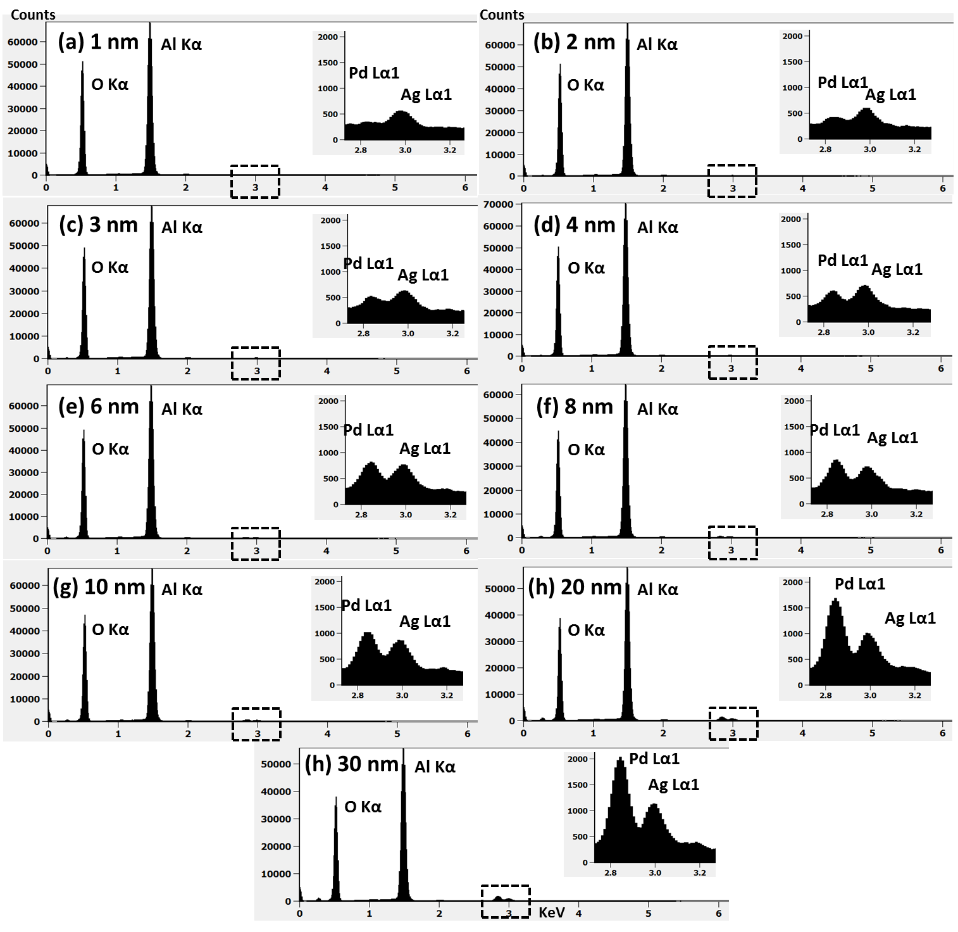
**

**Figure S24:** (a) – (h) EDS spectra the samples shown in Fig. S16. The intensity of the peaks corresponds to the amount of element. Insets highlight the Pd and Ag peaks namely Pd Lα1 at 2.836 keV and Ag Lα1 at 2.981 keV respectively.

**Table S1:** Summary of RMS roughness (Rq) and surface area ratio (SAR) of Pd-Ag alloy NPs on sapphire (0001) with the constant total thickness 15 nm and distinct compositions (Pd_0.25_Ag_0.75_, Pd_0.5_Ag_0.5_ and Pd_0.75_Ag_0.25_) annealed at identical environment i.e. between 400 and 900 °C for 120 s.

| **Temperature [°C]** | **Compositions** | | | | | |
| --- | --- | --- | --- | --- | --- | --- |
|  | **Pd_0.25_Ag_0.75_** | | **Pd_0.5_Ag_0.5_** | | **Pd_0.75_Ag_0.25_** | |
|  | **Rq [nm]** | **SAR [%]** | **Rq [nm]** | **SAR [%]** | **Rq [nm]** | **SAR [%]** |
| **400** | 2.81 | 0.74 | 3.58 | 0.85 | 11.26 | 0.77 |
| **500** | 6.18 | 2.22 | 9.70 | 2.25 | 8.34 | 0.93 |
| **600** | 13.03 | 3.93 | 15.37 | 3.83 | 11.93 | 1.46 |
| **700** | 20.17 | 7.58 | 26.49 | 10.59 | 19.12 | 3.41 |
| **800** | 20.33 | 8.59 | 28.08 | 13.02 | 26.57 | 9.02 |
| **900** | 20.86 | 10.40 | 23.28 | 8.44 | 30.04 | 10.32 |

**Table S2:** Summary of average height (AH), lateral diameter (LD) and average density (AD) of the Pd-Ag alloy NPs fabricated between 700 and 900 C for 120 s with 15 nm total thickness consisting Pd and Ag in a ratio of Pd_0.25_Ag_0.75_.

| **Temperature [ºC]** | **AH [nm]** | **LD [nm]** | **AD [× 10^7^ cm^-2^]** |
| --- | --- | --- | --- |
| **700** | 54.21 | 264.36 | 0.99 |
| **800** | 56.21 | 209.45 | 1.07 |
| **900** | 50.15 | 178.15 | 1.13 |

**Table S3:** Numerical summary of average reflectance of Pd-Ag alloy NPs on sapphire (0001) with constant total thickness 15 nm and distinct compositions (Pd_0.25_Ag_0.75_, Pd_0.5_Ag_0.5_ and Pd_0.75_Ag_0.25_) annealed between 400 and 900 °C for 120 s.

| **Temperature [°C]** | **Compositions** | | |
| --- | --- | --- | --- |
|  | **Pd_0.25_Ag_0.75_** | **Pd_0.5_Ag_0.5_** | **Pd_0.75_Ag_0.25_** |
|  | **Ref [%]** | **Ref [%]** | **Ref [%]** |
| **Bare** | 7.781 | 7.805 | 7.724 |
| **400** | 26.13 | 29.638 | 34.517 |
| **500** | 26.78 | 23.363 | 32.949 |
| **600** | 22.165 | 20.688 | 24.976 |
| **700** | 15.660 | 14.352 | 18.949 |
| **800** | 10.566 | 9.712 | 12.892 |
| **900** | 8.510 | 5.879 | 11.013 |

**Table S4:** Summary of Raman intensity and shift of Pd-Ag alloy NPs on sapphire (0001) fabricated between 400 and 900 °C for 120 s with total thickness of 15 nm and composition variation as depicted in table.

| **Temperature [°C]** | **Compositions** | | | | | |  |
| --- | --- | --- | --- | --- | --- | --- | --- |
|  | **Pd_0.25_Ag_0.75_** | | **Pd_0.5_Ag_0.5_** | | **Pd_0.75_Ag_0.25_** | |  |
|  | **Intensity [a.u.]** | **Shift**  **[cm^-1^]** | **Intensity [a.u.]** | **Shift**  **[cm^-1^]** | **Intensity [a.u.]** | **Shift**  **[cm^-1^]** | |
| **Bare** | 2722.315 | 417.1908 | 2630.863 | 417.221 | 2730.863 | 417.210 | |
| **400** | 641.80 | 417.102 | 448.174 | 417.029 | 249.330 | 417.271 | |
| **500** | 566.868 | 417.214 | 610.969 | 417.227 | 317.826 | 417.111 | |
| **600** | 785.915 | 417.155 | 740.632 | 417.129 | 521.093 | 417.087 | |
| **700** | 1213.418 | 417.163 | 1101.244 | 417.088 | 815.603 | 417.168 | |
| **800** | 1703.488 | 417.112 | 1521.979 | 417.137 | 1244.484 | 417.032 | |
| **900** | 2235.315 | 417.176 | 1583.034 | 417.151 | 1366.062 | 417.093 | |

**Table S5:** Summary of Rq and SAR of Pd-Ag alloy NPs fabricated at 850 ºC for 120 s with various total thickness and composition as depicted in table.

| **Thickness [nm]** | **Compositions** | | | | | |
| --- | --- | --- | --- | --- | --- | --- |
|  | **Pd_0.25_Ag_0.75_** | | **Pd_0.5_Ag_0.5_** | | **Pd_0.75_Ag_0.25_** | |
|  | **Rq [nm]** | **SAR [%]** | **Rq [nm]** | **SAR [%]** | **Rq [nm]** | **SAR [%]** |
| **1** | 1.02 | 1.12 | 0.58 | 0.32 | 1.53 | 1.84 |
| **2** | 1.15 | 1.35 | 3.09 | 4.39 | 3.94 | 5.72 |
| **3** | 5.45 | 5.62 | 6.35 | 10.07 | 9.48 | 11.54 |
| **4** | 6.91 | 8.03 | 9.48 | 10.57 | 12.56 | 14.21 |
| **6** | 11.07 | 8.84 | 13.78 | 10.80 | 17.03 | 12.19 |
| **8** | 14.42 | 11.38 | 14.75 | 10.84 | 18.73 | 11.51 |
| **10** | 13.57 | 9.23 | 17.03 | 11.06 | 20.88 | 10.95 |
| **20** | 17.64 | 9.75 | 26.43 | 10.24 | 28.27 | 14.44 |
| **30** | 30.97 | 8.28 | 33.47 | 11.20 | 47.10 | 12.99 |

**Table S6:** Summary of average height (AH), lateral diameter (LD) and average density (AD) of the Pd-Ag alloy NPs fabricated at 850 ºC for 120 s with total thickness variation between 1 and 30 nm consisting Pd and Ag in a ratio of Pd_0.25_Ag_0.75_.

| **Thickness [nm]** | **AH [nm]** | **LD [nm]** | **AD [× 10^10^ cm^-2^]** |
| --- | --- | --- | --- |
| **1** | 2.81 | 17.27 | 15.68 |
| **2** | 3.48 | 25.13 | 14.72 |
| **3** | 15.68 | 68.66 | 1.1 |
| **4** | 20.37 | 80.82 | 0.41 |
| **6** | 31.23 | 117.78 | 0.288 |
| **8** | 40.39 | 129.84 | 0.254 |
| **10** | 42.73 | 137.50 | 0.186 |
| **20** | 53.92 | 170.34 | 0.130 |
| **30** | 111.56 | 303.03 | 0.024 |

**Table S7:** Summary of average reflectance based on the reflectance spectra between 300 and 1100 nm of Pd-Ag alloy nanostructures on sapphire (0001) with various thickness between 1 and 30 nm with distinct composition of Pd and Ag.

| **Thickness [nm]** | **Compositions** | | |
| --- | --- | --- | --- |
|  | **Pd_0.25_Ag_0.75_** | **Pd_0.5_Ag_0.5_** | **Pd_0.75_Ag_0.25_** |
|  | **Ref [%]** | **Ref [%]** | **Ref [%]** |
| **Bare** | 7.517 | 7.763 | 7.741 |
| **1** | 8.250 | 6.704 | 8.161 |
| **2** | 7.742 | 7.619 | 8.403 |
| **3** | 8.295 | 7.363 | 8.572 |
| **4** | 8.084 | 8.454 | 8.671 |
| **6** | 8.059 | 5.932 | 9.425 |
| **8** | 8.391 | 4.565 | 10.809 |
| **10** | 4.852 | 9.168 | 9.757 |
| **20** | 9.739 | 8.853 | 13.171 |
| **30** | 8.746 | 7.507 | 6.749 |

**Table S8:** Numerical summary of Raman peak intensity and position probed by A_1g_ vibration mode in each sets of samples with various thickness and composition of bilayers, annealed at fixed 850 ºC for 120 s.

| **Thickness [nm]** | **Compositions** | | | | | | |
| --- | --- | --- | --- | --- | --- | --- | --- |
|  | **Pd_0.25_Ag_0.75_** | | **Pd_0.5_Ag_0.5_** | | **Pd_0.75_Ag_0.25_** | |  |
|  | **Intensity [a.u.]** | **Shift**  **[cm^-1^]** | **Intensity [a.u.]** | **Shift**  **[cm^-1^]** | **Intensity [a.u.]** | **Shift [cm^-1^]** | |
| **Bare** | 2736.57 | 419.60 | 2544.22 | 419.37 | 2515.59 | 419.26 | |
| **1** | 2678.19 | 419.53 | 2545.39 | 419.39 | 2552.41 | 419.27 | |
| **2** | 2679.58 | 419.47 | 2329.69 | 419.22 | 2410.35 | 419.25 | |
| **3** | 2556.90 | 419.54 | 2409.33 | 419.31 | 2359.37 | 419.28 | |
| **4** | 2520.44 | 419.53 | 2402.18 | 419.36 | 2376.81 | 418.15 | |
| **6** | 2431.74 | 419.50 | 2120.58 | 419.32 | 1604.38 | 419.25 | |
| **8** | 2161.55 | 419.47 | 1656.71 | 419.36 | 1338.03 | 419.29 | |
| **10** | 2186.57 | 419.47 | 1709.73 | 419.32 | 1480.15 | 419.25 | |
| **20** | 1762.23 | 419.41 | 1440.70 | 419.27 | 1105.61 | 419.14 | |
| **30** | 1934.50 | 419.36 | 1466.56 | 418.05 | 1235.91 | 419.22 | |
